# Clinical predictors of *BRCA1/2* P/LP variants for high-risk breast cancer patients in China: *HBRCA-risk prediction*

**DOI:** 10.3389/fonc.2026.1779548

**Published:** 2026-06-02

**Authors:** Wei Gu, Chengyin Xu, Jinzhen Fu, Huijun Lei, Najeeb Ullah Khan, Ruijiao Lei, Xukai Chen, Xiao-jia Wang, Tianhui Chen

**Affiliations:** 1 Postgraduate Training Base Alliance of Wenzhou Medical University (Zhejiang Cancer Hospital), Wenzhou, China; 2 Department of Cancer Prevention, Zhejiang Cancer Hospital, Hangzhou, China; 3 Hangzhou Institute of Medicine (HIM), Chinese Academy of Sciences, Hangzhou, China; 4 Institute of Biotechnology and Genetic Engineering, The University of Agriculture Peshawar, Peshawar, Pakistan; 5 Department of Breast Medical Oncology, Zhejiang Cancer Hospital, Hangzhou, China

**Keywords:** BRCA1/2, breast cancer, China, gene testing, risk prediction

## Abstract

**Background:**

Germline *BRCA1/2* pathogenic or likely pathogenic (P/LP) variant identification is critical for guiding surgical and systemic therapy in breast cancer. However, prediction tools developed in high-risk cohorts remain limited, hindering large-scale adoption in clinical pathways in China.

**Methods:**

We included 1,204 high-risk breast cancer patients during 2017–2021 from Zhejiang Cancer Hospital, eastern China. Clinical data were collected and blood samples underwent targeted NGS for *BRCA1/2*, with variants classified by ClinVar and the American College of Medical Genetics and Genomics-Association for Molecular Pathology (ACMG-AMP). Predictors (histology, molecular subtype, age, and family history) were included with missing data imputed using Multiple Imputation by Chained Equations (MICE). We developed a multivariable logistic regression model to predict P/LP carrier status and evaluated its performance across imputed datasets with bootstrap internal validation.

**Results:**

In 1,204 high-risk Chinese breast cancer patients, *BRCA1/2* P/LP variants were detected in 102 (8.5%), with strong associations for triple-negative breast cancer (TNBC) (55.9%), invasive ductal carcinoma (IDC) (94.1%), and family history, while older age reduced risk. The final model incorporated histology, molecular subtype, age, and family history. It achieved good discrimination and acceptable calibration, with a low Brier score. The area under the receiver operating characteristic curve (AUC) was 0.758, the Hosmer–Lemeshow (HL) *P* value was 0.349, and the Brier score was 0.071. Sensitivity, stratified, and bootstrap validation (500 resamples, calibration error 0.007) confirmed robustness. Decision Curve Analysis (DCA) demonstrated clear net clinical benefit over test-all and test-none strategies.

**Conclusions:**

We developed a clinicopathology-based model from a high-risk Chinese breast cancer cohort to predict *BRCA1/2* P/LP carrier probability, which was the large high-risk clinical cohort with information of *BRCA1/2* variants and clinical characteristics in mainland China. It supported clinical implementation by extending testing beyond current guidelines and optimizing the use of limited genetic resources.

## 1 Introduction

Identifying germline *BRCA1/2* variants in women with breast cancer informs surgical choices, systemic therapy, and cascade testing for relatives, and now directly guides poly ADP-ribose polymerase (PARP) inhibitor use across disease stages. Landmark trials show clinically meaningful benefit with olaparib in *BRCA1/2* early and metastatic disease, for example, OlympiA improved invasive disease-free survival, overall survival, and progression-free survival, underscoring the treatment consequence of knowing carrier status (1, 2). *BRCA1/2* variants are found in about 13-17% of high-risk breast cancer patients, who would benefit mostly from genetic testing (3–5).

Professional guidelines have broadened access to testing. The American Society of Clinical Oncology – Society of Surgical Oncology (ASCO-SSO) 2024 guideline recommends offering *BRCA1/2* testing to all newly diagnosed patients ≤65 years and to selected patients >65 years based on personal/family history, ancestry, or PARP candidacy (6). In primary care, the United States Preventive Services Task Force (USPSTF) advises using a brief familial risk-assessment tool to trigger counseling or testing, especially in healthcare settings where genetics services are limited or inconsistently available (7). In China, current guidelines recommend *BRCA1/2* testing for patients with a high likelihood of hereditary risk, such as early-onset disease, multiple primaries, triple-negative breast cancer (TNBC), male breast cancer, or strong family history, but routine testing of all newly diagnosed patients has not yet been adopted (8, 9). Despite guideline recommendations, germline testing remains underused in real-world practice. A large cohort study has shown that only around 31%–56% of eligible patients undergo testing, with marked variation by tumor subtype and healthcare setting, leaving many actionable carriers unidentified (10).

To triage testing efficiently, especially for patients not covered by guideline rules, clinical tools derived from a large high-risk cohort with both variants and clinical information are needed that run on routinely available characteristics. Established predictors (e.g., BRCAPRO, BOADICEA, Manchester) remain important, but they may require detailed pedigree information and may show population-dependent calibration, which has prompted efforts to develop population-specific updates and pathology-informed adaptations (11, 12). Studies in Asian populations, including Chinese cohorts, have similarly suggested that locally calibrated and pathology-informed models may improve risk prediction (13). Nevertheless, limited prediction models have been developed in large high-risk clinic cohorts of already diagnosed patients that incorporate both clinicopathological characteristics and genetic outcomes, although this setting closely reflects routine referral and counseling workflows (13). There is an urgent need for a clinic-based carrier-probability model to support guideline-recommended testing and triage counseling when genetic services are limited.

We developed and internally validated a clinicopathology-based logistic regression model to estimate the probability of carrying *BRCA1/2* pathogenic or likely pathogenic (P/LP) variants in a large cohort diagnosed with high-risk breast cancer from Zhejiang Cancer Hospital in eastern China, with genetic and clinical data. The predictors included age at diagnosis, histological subtype, molecular subtype, and family history. Model performance was evaluated in terms of discrimination, assessed by the area under the receiver operating characteristic curve (AUC), calibration, assessed by the Hosmer–Lemeshow test (HL), and bootstrap calibration, and overall prediction error, assessed by the Brier score. Internal validation of the model was conducted with 500 bootstrap resamples. Sensitivity, stratified, and Firth-corrected analyses were conducted to assess robustness and reduce potential bias. Clinical utility was examined with decision curve analysis (DCA) against test-all and test-none strategies. The final model was presented as a nomogram to facilitate *BRCA1/2* testing decisions and support clinical risk stratification and resource use in practice.

## 2 Materials and methods

### 2.1 Study participants

**Figure 1:**
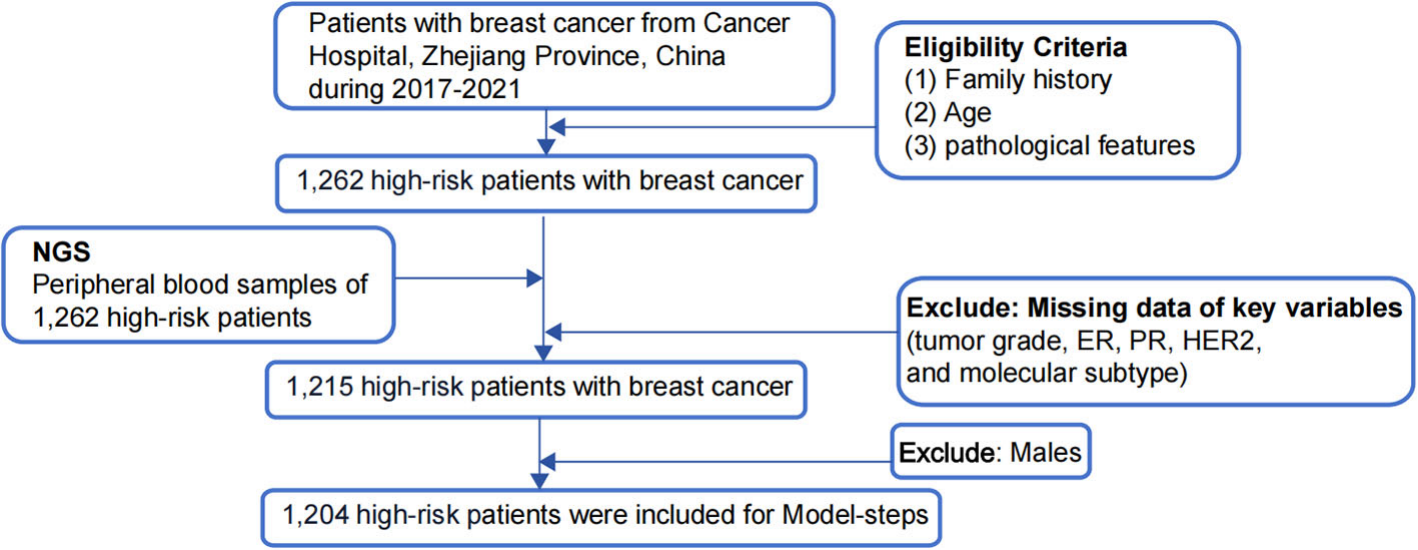
Flowchart of patient inclusion criteria.

The research involved 1,262 high-risk breast cancer patients diagnosed from 2017 to 2021 at Zhejiang Cancer Hospital, Zhejiang Province, China, as illustrated in **Figure 1**. Eligibility was defined as meeting any of the following criteria: (1) early-onset breast cancer diagnosed at ≤45 years; (2) ≥2 primary tumors diagnosed at ≤50 years; (3) family history features, defined as breast cancer diagnosed at ≤50 years with one or more first-degree relatives with breast cancer, any age with ≥2 first-degree relatives with breast cancer diagnosed at ≤50 years, at least two first-degree relatives with pancreatic or prostate cancer (Gleason score ≥7), or a first-degree relative with male breast cancer; (4) TNBC diagnosed at ≤60 years; and (5) male breast cancer. To ensure clinical generalizability, 47 patients were excluded due to simultaneous missing data for key variables (tumor grade, individual receptor status, and molecular subtype), and male patients were also excluded since current guidelines already recommend genetic testing for all male breast cancer cases. Finally, 1,204 patients were included for further analysis. Clinical data were retrieved from the electronic medical record system.

### 2.2 Genomic DNA extraction and variant interpretation

Genomic DNA extracted from peripheral blood was used for library preparation with the NEBNext Ultra II DNA Library Prep Kit. Target enrichment was then performed with a custom 98-gene hereditary cancer panel (**Supplementary Table 1**). Sequencing was carried out on the Illumina HiSeq X Ten platform using 2 × 150 bp paired-end reads. Raw data were first assessed with FastQC and MultiQC. Adapter trimming was performed with Trim Galore. Reads were aligned to the GRCh38 reference genome using BWA-MEM (v0.7.17), and post-alignment processing was completed with fgbio and GATK (v4.2). Variants were called with both GATK HaplotypeCaller and FreeBayes, and were then filtered and annotated using ANNOVAR and Ensembl VEP. Variant analysis in this study focused on SNVs and small indels. Detailed per-sample sequencing coverage and quality statistics are provided in **Supplementary Table 2**.

Variant interpretation mainly followed the ACMG-AMP 2015 framework. Evidence considered for classification included ClinVar (accessed June 25, 2025), population frequency data from gnomAD EAS, and in silico predictions from SIFT, PolyPhen-2, MutationTaster, and CADD. *BRCA1/2* variants were reported using HGVS nomenclature based on the MANE Select transcripts NM_007294.4 and NM_000059.4. Final classifications were assigned as pathogenic, likely pathogenic, variants of uncertain significance (VUS), likely benign, or benign according to the overall ACMG-AMP evidence profile. Variants with insufficient or conflicting evidence for either pathogenic or benign classification were retained as VUS and were not treated as pathogenic findings in the main clinical interpretation.

### 2.3 Statistical analysis

Candidate predictors were first evaluated in univariate analyses, with multiple testing controlled by the Benjamini–Hochberg procedure. The outcome was *BRCA1/2* P/LP carrier status, given its direct clinical relevance for genetic counseling, testing interpretation, and subsequent management. Details of *BRCA1/2* P/LP variants were shown in **Supplementary Table 3**. Variables showing statistical significance and supported by clinical relevance were retained. Owing to strong correlations between molecular subtype and individual receptor status, including estrogen receptor (ER), progesterone receptor (PR), human epidermal growth factor receptor 2 (HER2), only the molecular subtype was preserved to avoid redundancy. Missing data were handled using multiple imputation by chained equations (MICE), generating 300 completed datasets. Model fitting and performance evaluation were performed separately within each imputed dataset. Regression coefficients and standard errors were combined using Rubin’s rules, whereas AUC and calibration statistics were calculated within each imputed dataset and averaged across imputations. For each imputed dataset, the AUC and its standard error were estimated using the DeLong method. For visualization, 30 imputed datasets were randomly selected for plotting. The final multivariable logistic regression model incorporated four predictors: histological subtype, molecular subtype, age at diagnosis, and family history. Robustness was assessed through stratified analyses by invasive ductal carcinoma (IDC) status, sensitivity analyses reintroducing grade, and complete-case analyses using both standard logistic regression and the Firth correction. Across all approaches, results remained consistent. Model performance was quantified in terms of discrimination, calibration, and overall accuracy. Discrimination was assessed by the AUC with 95% confidence intervals, calibration by the HL test and bootstrap calibration curves (B = 500), and overall accuracy by the Brier score. Internal validation with 500 bootstrap resamples further demonstrated the stability of the model. All statistical analyses were performed in R (version 4.5.0), including mice, pROC, ResourceSelection, rms, rmda, ggplot2, dplyr, broom, and logistf packages. *P* value <0.05 (two-tailed) was considered statistically significant.

## 3 Results

### 3.1 Clinicopathologic characteristics

The clinicopathologic characteristics of our cohort of 1,204 high-risk breast cancer patients covering *BRCA1*/*2* testing were presented in **Table 1**. *BRCA1/2* P/LP variants occurred in 102 cases (8.5%). A summary of *BRCA1/2* variant distribution is provided in **Supplementary Figure 1**. Bilateral breast cancer, histological subtype, tumor grade, ER, PR, HER2, molecular subtype and family history were significantly associated with carrier status. Among molecular subtypes, TNBC showed the strongest association with *BRCA1/2* carrier status, with carriers more frequently classified as TNBC (55.9%, *P* < 0.001).

**Table 1:** Baseline characteristics and univariable associations with *BRCA1/2* carrier status.

Characteristics		*BRCA*^+^ (n = 102)	*BRCA*^-^ (n = 1,102)	*P*
Bilateral	YES	8 (7.84%)	1,002 (90.93%)	<0.001^a^
	NO	94 (92.16%)	100 (9.07%)	
Histological	IDC	96 (94.12%)	978 (88.75%)	0.014^b^
	ILC	0 (0.00%)	27 (2.45%)	
	situ	2 (1.96%)	32 (2.90%)	
	OtherIC	0 (0.00%)	60 (5.44%)	
	NA	4 (3.92%)	5 (0.45%)	
Grade	I	1 (0.98%)	22 (2.00%)	0.024^b^
	II	28 (27.45%)	368 (33.39%)	
	III	44 (43.14%)	342 (31.01%)	
	NA	29 (28.43%)	370 (33.58%)	
ER	positive	38 (37.25%)	659 (59.80%)	<0.001^a^
	negative	63 (61.76%)	435 (39.47%)	
	NA	1 (0.98%)	8 (0.73%)	
PR	positive	35 (34.31%)	592 (53.72%)	<0.001^a^
	negative	66 (64.71%)	500 (45.37%)	
	NA	1 (0.98%)	10 (0.91%)	
Her2	positive	7 (6.86%)	282 (25.59%)	<0.001^a^
	negative	87 (85.29%)	755 (68.51%)	
	NA	8 (7.84%)	65 (5.90%)	
Molecular				<0.001^a^
	Her2 overexpress	2 (1.96%)	124 (11.25%)	<0.01^a^
	Luminal A	30 (29.41%)	478 (43.38%)	<0.01^a^
	Luminal B	5 (4.90%)	158 (14.34%)	<0.01^a^
	TNBC	57 (55.88%)	277 (25.14%)	<0.001^a^
	NA	8 (7.84%)	65 (5.90%)	
Distal	YES	7 (6.86%)	69 (6.26%)	0.811^a^
	NO	95 (93.14%)	1,033 (93.74%)	
Age at diagnosed	Mean	40.5	41.8	0.504^c^
Family history	YES	57 (55.88%)	420 (38.11%)	<0.001^a^
	NO	45 (44.12%)	682 (61.89%)	
BMI	< 18.5	7 (6.86%)	55 (4.99%)	0.593^b^
	18.5-24	52 (50.98%)	656 (59.53%)	
	24-28	22 (21.57%)	225 (20.42%)	
	≥ 28	4 (3.92%)	50 (4.54%)	
	NA	17 (16.67%)	116 (10.53%)	
Age at menarche	10-16	74 (72.55%)	833 (75.59%)	0.934^a^
	>16	8 (7.84%)	93 (8.44%)	
	NA	20 (19.61%)	176 (15.97%)	
Menopausal status	Postmenopausal	20 (19.61%)	180 (16.33%)	0.252^a^
	Premenopausal	65 (63.73%)	795 (72.14%)	
	NA	17 (16.67%)	127 (11.52%)	
Number of births	Mean	5.6 (5.49%)	1.2 (0.11%)	0.703^c^

a chi-squared test; ^b^Fisher’s exact test; ^c^Wilcoxon; ER, estrogen receptor; PR, progesterone receptor; HER2, human epidermal growth factor receptor 2; IDC, Invasive Ductal Carcinoma; ILC, invasive lobular carcinoma; situ, carcinoma in Situ; Other IC, other invasive carcinoma; TNBC, triple negative breast cancer.

In terms of histological subtype, IDC was more common among carriers than non-carriers (94.12% vs. 88.75%). The lower frequency of bilateral breast cancer among *BRCA1/2* carriers in our cohort may be a result of the relatively short follow-up period (2017–2021).

### 3.2 Development of a HBRCA-risk prediction model

**Figure 2:**
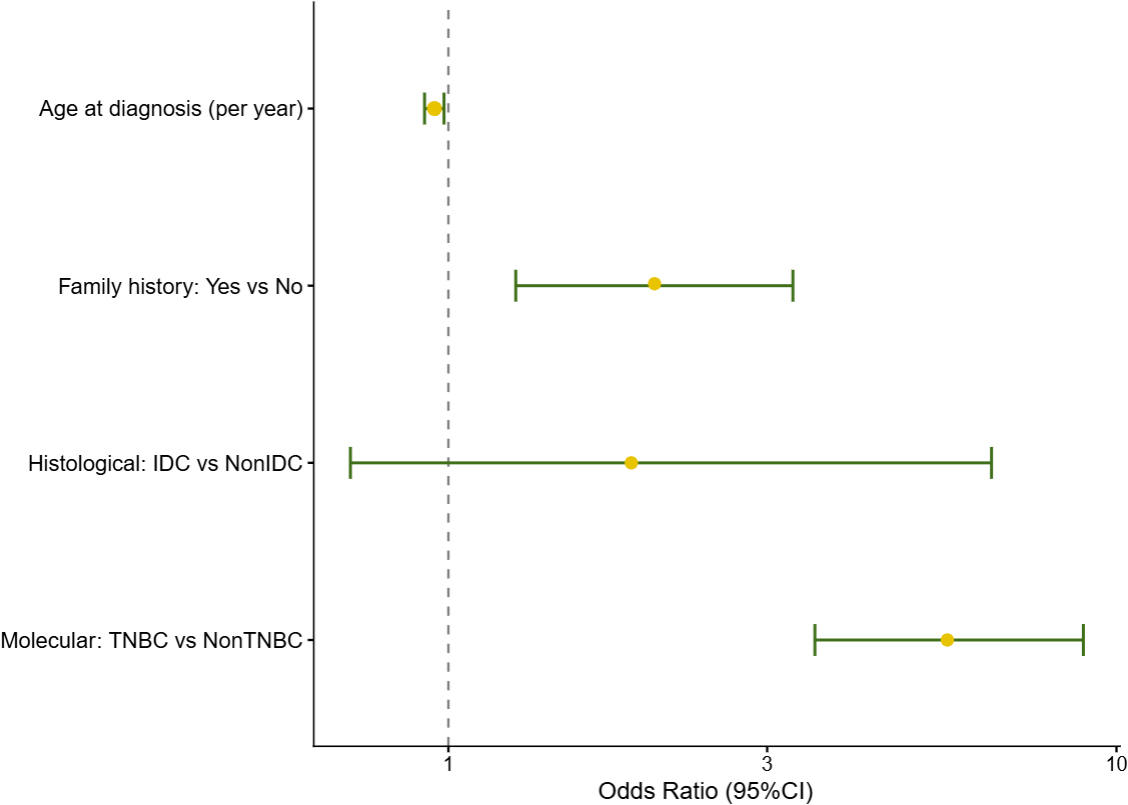
Forest plot of adjusted odds ratios for *BRCA1/2* P/LP carrier status from multivariable logistic regression (95% CI).

As shown in **Figure 2**, the final multivariable logistic regression model included four predictors, histological subtype, molecular subtype, age at diagnosis, and family history. IDC (OR 5.74, 95% CI 1.38-23.95), TNBC subtype (OR 5.41, 95% CI 3.45-8.49), and family history (OR 3.59, 95% CI 2.25-5.75) were significantly associated with *BRCA1/2* P/LP variant carrier status, whereas the elder age at diagnosis was inversely associated (OR 0.96 per year, 95% CI 0.93-0.98).

Several sensitivity analyses confirmed the robustness of the findings. Across multiply imputed datasets, predictor distributions were largely stable (**Supplementary Figure 2**), with some variation in IDC-specific estimates. Stratified analyses by IDC versus non-IDC yielded consistent directions of association (**Supplementary Figure 3**). Tumor grade, although significant in univariable analysis, was not retained in the multivariable model. Its inclusion in sensitivity models did not improve discrimination compared with the primary model (DeLong test, *P* > 0.05).

**Figure 3:**
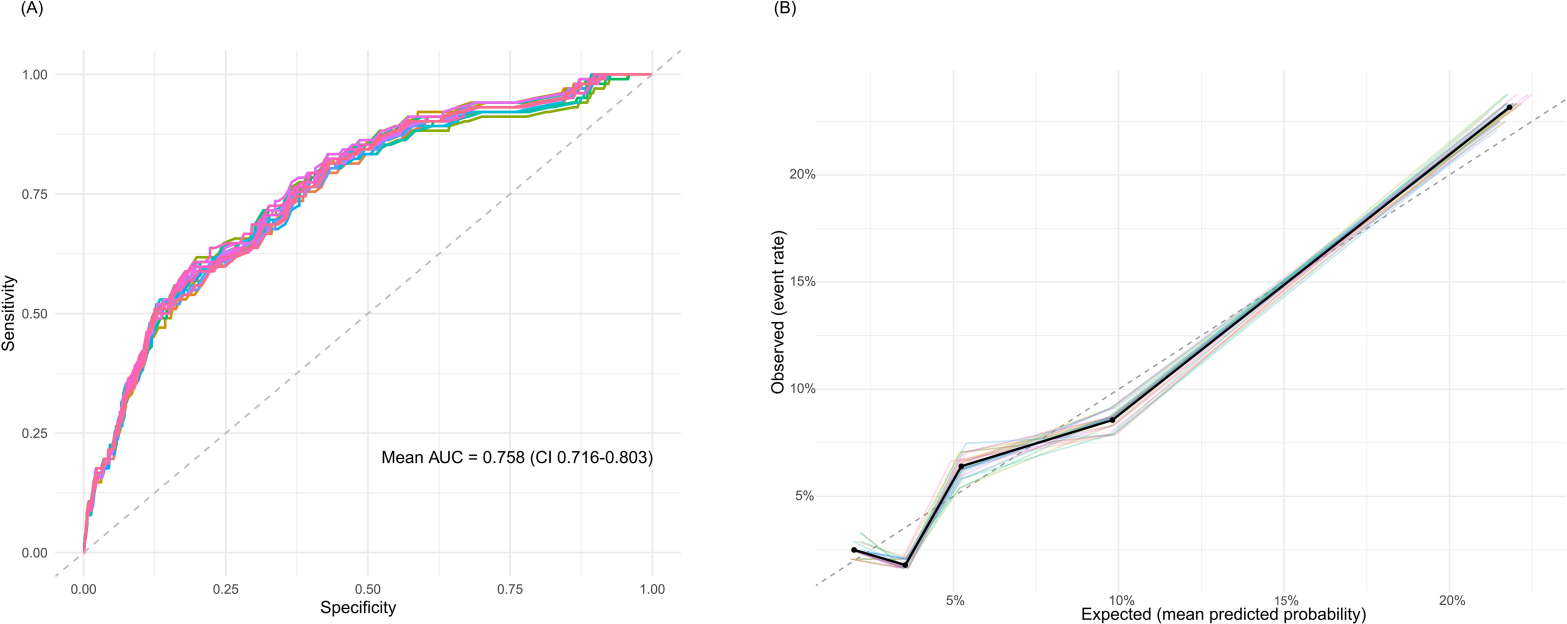
Model performance across 30 imputations (a random subset of the 300 total imputed datasets for visualization only) for predicting P/LP carrier status: **(A)** ROC curves and average AUC (0.758, 95% CI 0.716-0.803); **(B)** calibration plots with Hosmer–Lemeshow test *P* values (0.349, 0.0738-0.6370) and Brier score (0.0708 ± 0.0002).

**Figure 4:**
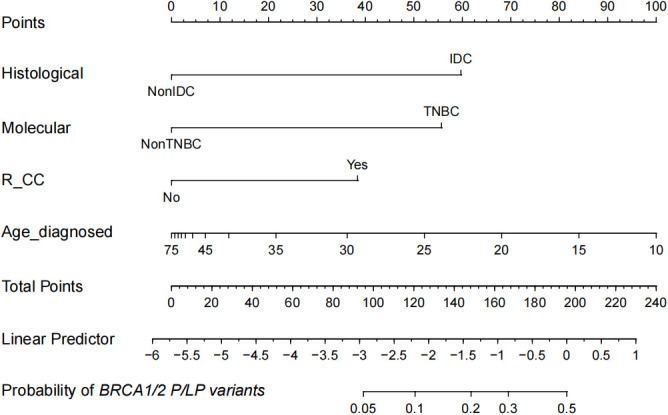
Nomogram based on the final multivariable model.

**Figure 5:**
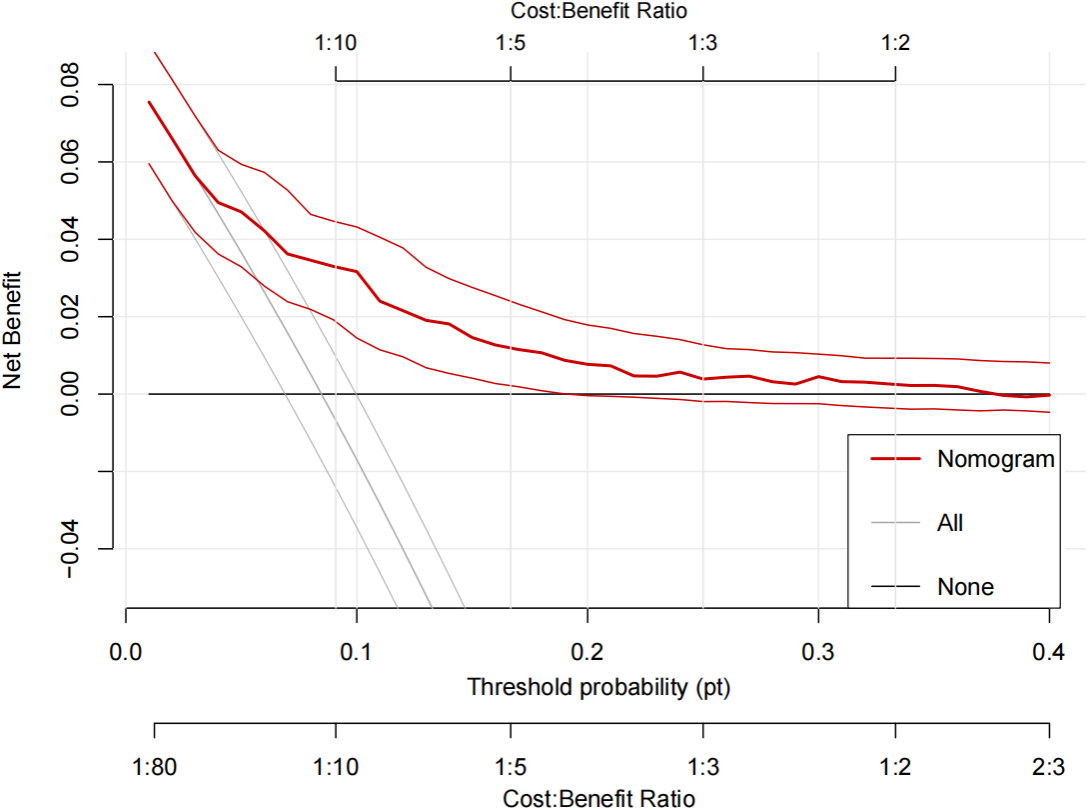
Decision curve analysis comparing net benefit of the nomogram for predicting P/LP carrier status with treat-all and treat-none strategies across threshold probabilities.

The final model, named the HBRCA-risk prediction model (*BRCA1/2* P/LP variants for high-risk breast cancer patients in China), demonstrated good discrimination, with an AUC of 0.758 (95% CI 0.716-0.803) (**Figure 3A**). The HL test indicated no significant lack of fit (median *P* = 0.349), and calibration plots showed close agreement between predicted and observed risks (**Figure 3B**). The mean Brier score was 0.071, supporting overall predictive accuracy. A nomogram incorporating the four final predictors was developed (**Figure 4**). The DCA curve indicated that the model provided greater net benefit than either the treat-all or treat-none strategy across clinically relevant threshold probabilities (approximately 5–30%; **Figure 5**). An additional analysis stratified by molecular subtype (TNBC vs non-TNBC) was performed using *HBRCA-risk prediction model*, and the corresponding results were shown in **Supplementary Figure 4**, the subgroup analyses were intended to provide descriptive insights into model performance rather than to test differences between groups, formal comparisons were not performed. The model performed better in the overall high-risk clinical cohort than in either molecular subtype-defined subgroup.

### 3.3 Internal validation of the model

**Figure 6:**
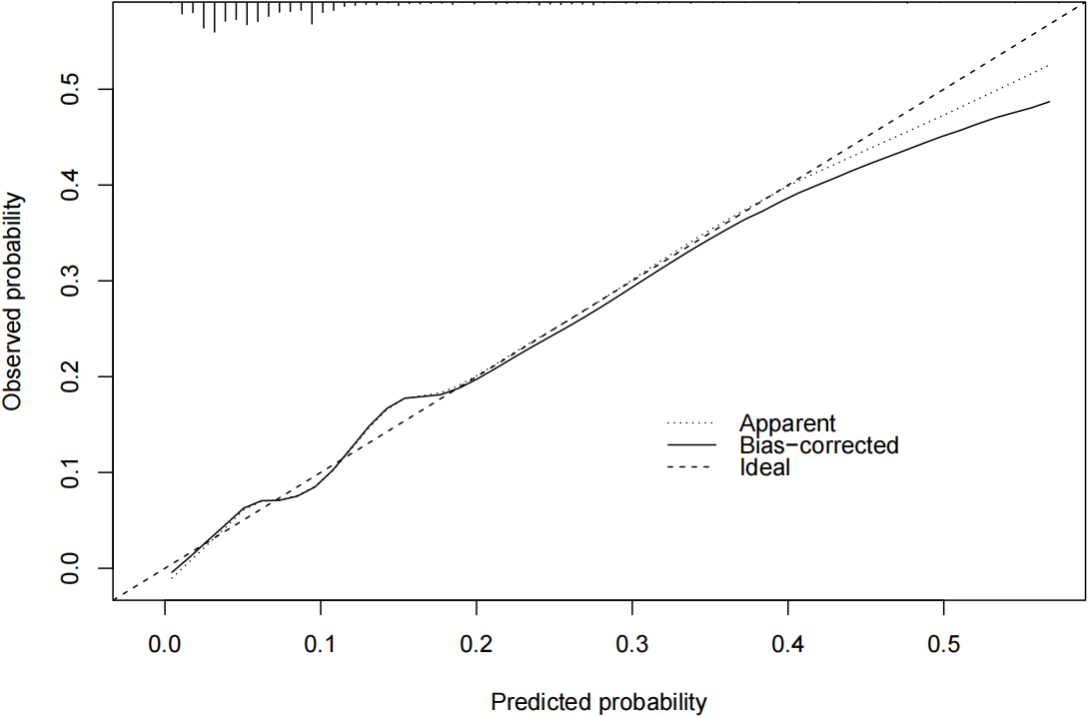
Calibration plot with bootstrap internal validation for predicting P/LP carrier status (apparent vs bias-corrected).

As shown in **Figure 6**, internal validation was performed using 500 bootstrap resamples. The optimism-corrected calibration curve showed close agreement with the ideal line, indicating good calibration of the model. The bootstrap-corrected AUC and Brier score were consistent with the apparent estimates, and the mean absolute calibration error was 0.007, indicating very good agreement between predicted and observed probabilities, thus further supporting the robustness and generalizability of the model.

## 4 Discussion

In this high-risk clinic cohort (n=1,204), we developed a clinic-based model to estimate the probability of carrying *BRCA1/2* P/LP variants using four routinely available predictors, including histologic subtype, molecular subtype, age at diagnosis, and family history. The model showed acceptable discrimination (AUC 0.758), good calibration, and low overall error (Brier 0.071). DCA indicated greater net clinical benefit than either the treat-all strategy or the treat-none strategy across threshold probabilities from 5% to 30%. These results suggested potential value for probability-guided triage of genetic testing in clinical practice.

Among patients with TNBC, the prevalence of germline *BRCA1/2* variants is commonly around 10–20% and a majority of tumors arising in *BRCA1* carriers display TNBC with 57–80% reported (14). Using molecular subtype rather than separate ER, PR, and HER2 markers may help reduce categorical redundancy while preserving bedside interpretability. This approach is also consistent with pathology-adjusted Manchester score (MSS3) updates, in which TNBC is explicitly weighted when estimating the 10% and 20% testing thresholds (12). Family history retained a strong association, consistent with guideline frameworks that place history-based risk assessment early in the genetic counseling and testing pathway (15). The inverse age effect aligned with the earlier onset typical of hereditary breast cancer (16). In real-world care, however, germline testing remains underused (17). By contrast, organized high-risk breast care pathways may achieve markedly higher testing uptake. For example, a recent nationwide mainstreaming study reported 77% of eligible patients with TNBC aged under 60 years underwent testing, 80% among those diagnosed at <40 years, and 67% overall among eligible patients, levels that were substantially higher than the population average (18).

Our findings regarding family history were consistent with evidence from Chinese cohorts, especially the Taiwanese series (19). In the Taiwanese cohort of 647 women, among the 241 individuals with both personal and family history, BOADICEA and BRCAPRO achieved an AUC of about 0.79. In the subset with available receptor data (n = 424), predictive performance in the TNBC subgroup was higher (AUC 0.74–0.80) compared to luminal or HER2 overexpressed subtypes (AUC 0.63–0.69). First, that suggested that in Chinese populations, models may perform better in patients with a family history and in more aggressive pathological subtypes. Second, among Chinese patients with TNBC, the detection rate of *BRCA1/2* variants was 14%, whereas in non-TNBC cases it was only 6.3% (20). Moreover, evidence from Chinese and other Asian cohorts indicated that prediction models generally achieve higher discrimination among individuals with a family history or with aggressive tumor phenotypes (20–22). Based on this evidence, we retained family history as a core predictor in our model and emphasized molecular subtype rather than individual receptor markers, which may improve predictive performance while preserving interpretability. In addition, the Taiwanese study focused on validating existing models rather than developing a model specifically for the Chinese population. Those models depend heavily on pedigree information and make limited use of routine clinical and pathological features, which constrains their applicability in broader clinical settings. In contrast, our model was developed using commonly available clinicopathologic predictors in a larger high-risk cohort, which may make it more applicable in clinical settings where detailed pedigree information is unavailable or incomplete. Importantly, our study included approximately twice as many participants as the Taiwanese cohort, which may further strengthen the stability of model development and support its broader clinical application in high-risk patients with breast cancer in China.

Unselected cohorts typically showed 5-6% *BRCA1/2* prevalence, whereas our high-risk clinic observed a higher carrier rate, this difference indicated that unselected cohorts may need substantially larger sample sizes to accumulate comparable events, which could directly affect model stability and calibration (23, 24). Second, large unselected Chinese breast cancer cohorts have consistently shown an association between TNBC and pathogenic *BRCA1/2* variant status, particularly for *BRCA1*, although these findings do not directly establish performance in high-risk settings, they supported the clinical relevance of TNBC as a predictor. This strengthened the rationale for its inclusion in *HBRCA-risk prediction*, while underscoring the need for validation in the high-risk population (23, 25). Third, timely access to germline testing and genetic counseling could be limited in routine practice (18, 26, 27), in this context, our *HBRCA-risk prediction*, a clinic risk tool, may help prioritize testing within high-risk referral pathways in the real-world setting.

Our model was expected to help to guide germline testing decisions for high-risk patients diagnosed with breast cancer for high-risk patients. In China, early implementation would most likely occur in high-risk referral clinics (e.g., TNBC, early-onset, positive family history) (28). Although TNBC is widely recognized as an indication for germline testing, in routine care, some patients remain hesitant to undergo testing despite meeting testing criteria (27). The uptake of testing could still be influenced by limited genetic knowledge, financial burden, concerns about possible adverse consequences, and policy-related factors (29, 30). In addition, for non-TNBC patients, decisions regarding *BRCA1/2* testing are often less directly supported by guidelines than in TNBC. This model could provide support by integrating routinely available clinical information, to help identify individuals more likely to benefit from testing (31). In this way, it may facilitate more informed decision-making in non-TNBC patients and support subsequent counseling, genetic testing decisions, and precision management. A practical and intuitive risk-assessment tool may help bridge the gap between guideline eligibility and real-world testing uptake by facilitating communication, improving patient understanding, and supporting next-step clinical decision-making. This model was developed for the overall high-risk breast cancer population in the genetic counseling setting, rather than specifically for either the TNBC or the non-TNBC subgroup. Performance of the model was better in the overall cohort than in the subtype-stratified analyses, consistent with its intended use in a population that more closely reflects real-world clinical practice. Developing the model in this enriched clinical setting may be an effective strategy. First, our cohort had a higher carrier rate (8.5%) than recent large unselected Chinese breast cancer cohorts, in which the prevalence was approximately 6% (32, 33), which may allow adequate carrier events for more stable estimation without requiring much larger samples (26). Second, our *HBRCA-risk prediction* is aligned with high-risk referral clinics in China. Although *BRCA1/2* testing is broadly recommended by guidelines, for example, ASCO-SSO 2024 recommends testing for patients ≤65 years and selected patients >65 years, uptake and service capacity could vary among different regions and hospitals (6).

This study had several strengths. First, we included a large cohort of 1,204 high-risk women with breast cancer from Zhejiang Cancer Hospital, Zhejiang Province, China, which helped support stable model estimation. Second, because the model was based only on routinely available clinicopathologic variables, it may be more feasible to implement in routine practice, particularly in regions and hospitals with limited clinical resources. Third, conducting the study in a high-risk Chinese population may further increase clinical relevance, since testing resources remain limited and carrier prevalence was higher in this group than in the unselected population. Thus, a probability-guided tool could provide meaningful support as a complement to existing guideline-based strategies. We also had some limitations. First, the single-center design and concentration of cases compromise the external validity of our findings and limit the direct extrapolation of our model to broader clinical settings across China. Second, the exclusion of 58 patients due to missing core variables and male breast cancer status may introduce a minor potential selection bias, which may restrict the generalizability of our model to breast cancer populations with limited clinical information. Third, while internal validation supported model discrimination and calibration, formal external validation remains needed to assess transportability, with modest recalibration where indicated. Therefore, to address these limitations and enhance the model’s clinical applicability, subsequent studies will enroll large, ethnically diverse samples from multiple clinical centers nationwide, and further optimize the model through targeted recalibration and subgroup-specific validation to support its safe and effective broad implementation in clinical practice.

In summary, this study developed an effective and clinic- precision model derived from a high-risk Chinese cohort of 1,204 women with breast cancer from Zhejiang Cancer Hospital, Zhejiang Province, eastern China. The model performed well with routinely available predictors, including histologic subtype, molecular subtype, age at diagnosis and family history. This model may help identify additional patients who could benefit from testing and thereby support more efficient use of limited genetic testing resources in routine practice.

## Data Availability

The data are available from the corresponding author on easonable request. Requests to access the data should be directed to Tianhui Chen chenth@zjcc.org.cn.
