# Supplementary figures and images for: Clinical predictors of BRCA1/2 P/LP variants for high-risk breast cancer patients in China: HBRCA-risk prediction
Source: Front Oncol. 2026 Jun 2;16:1779548. doi: 10.3389/fonc.2026.1779548 (PMC13268984; doi:10.3389/fonc.2026.1779548)

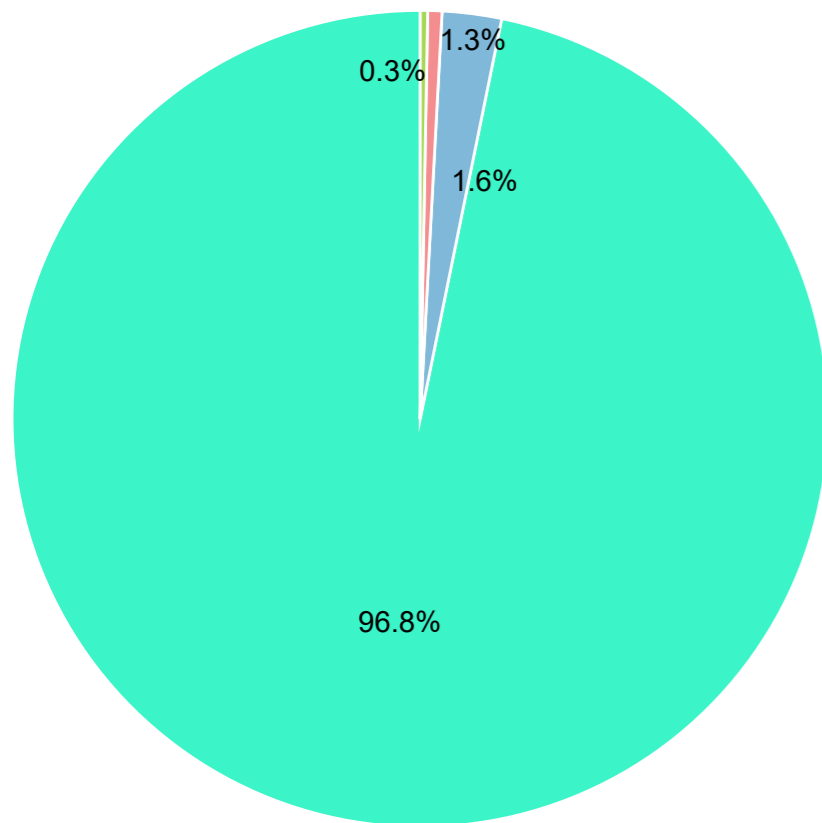

*BRCA 1*

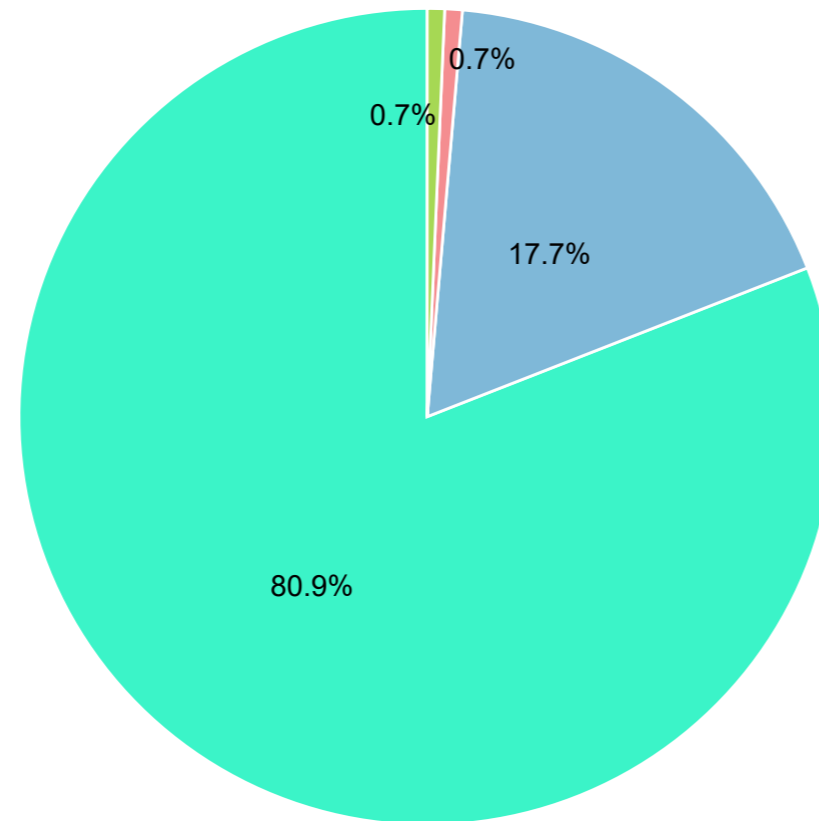

*BRCA 2*

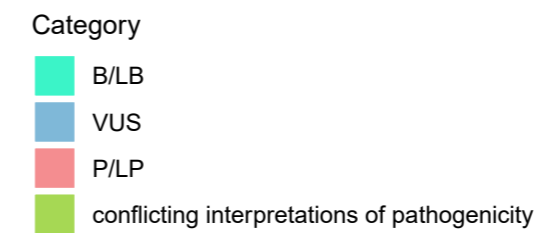

Supplement: Supplementary file 1 [file DataSheet1.pdf]

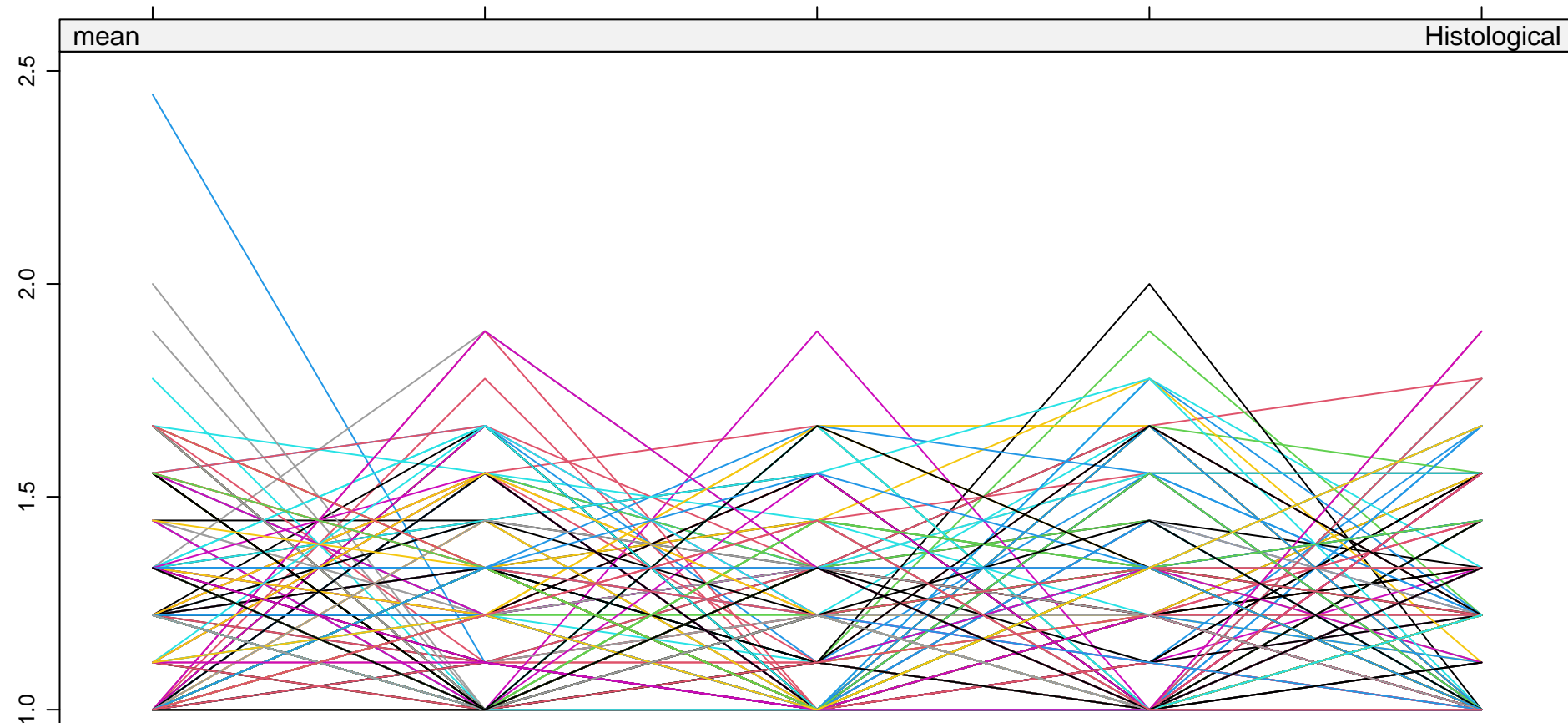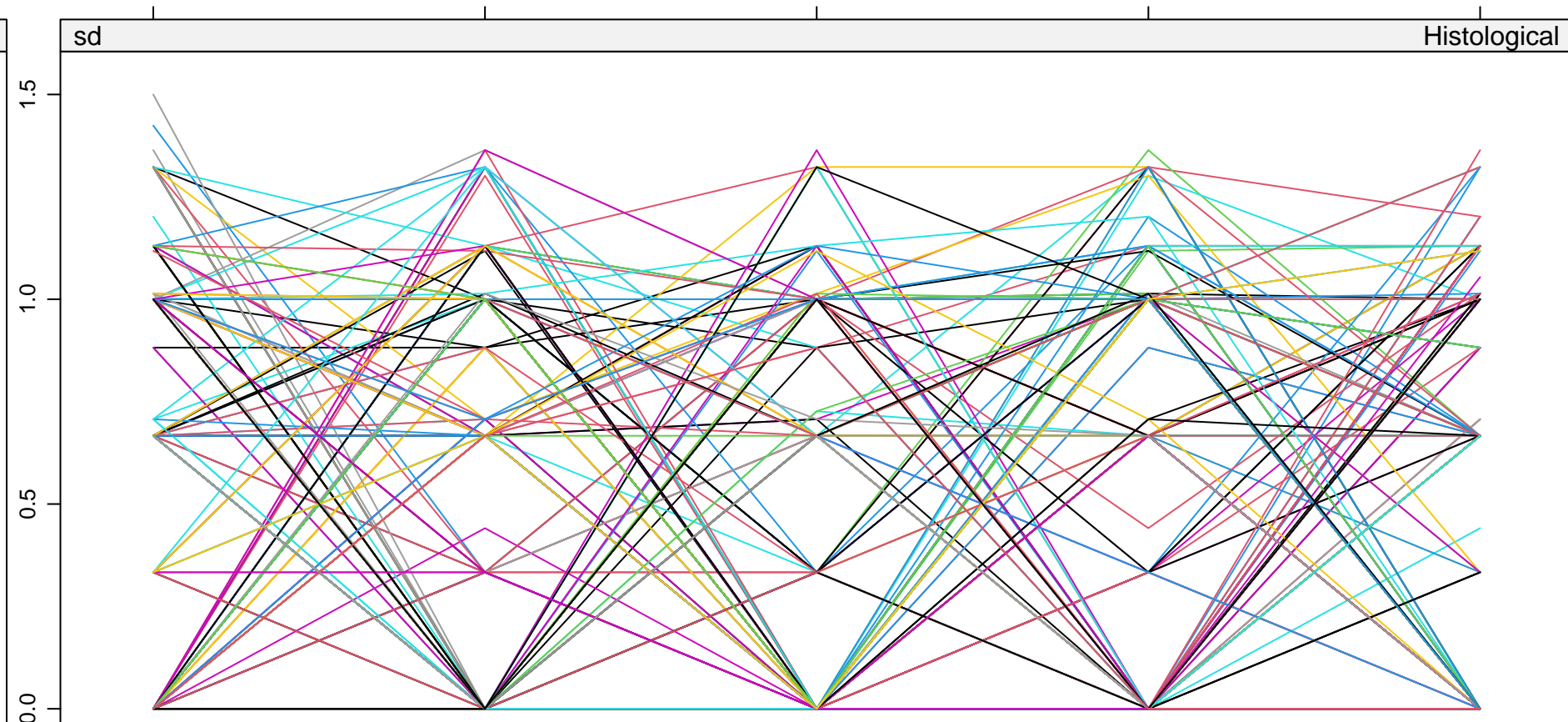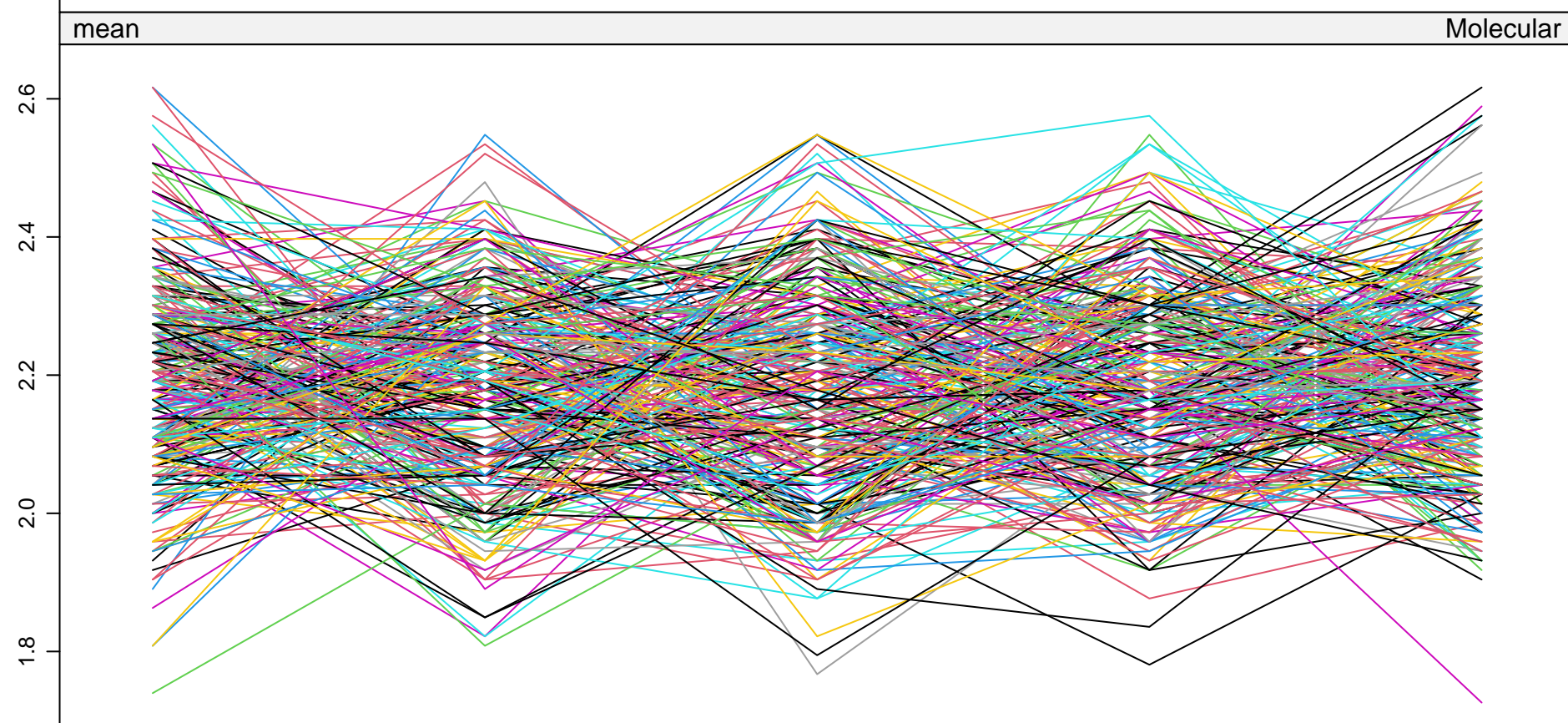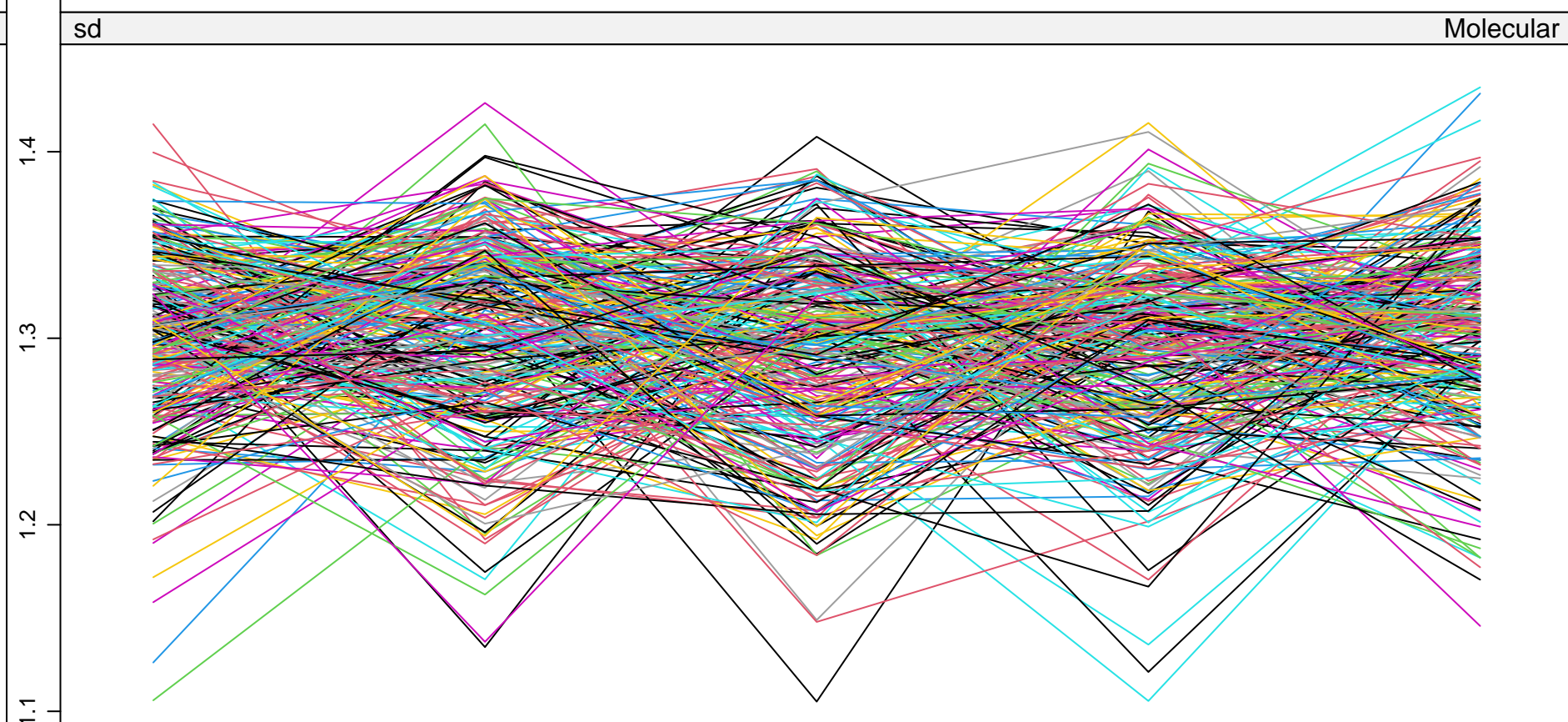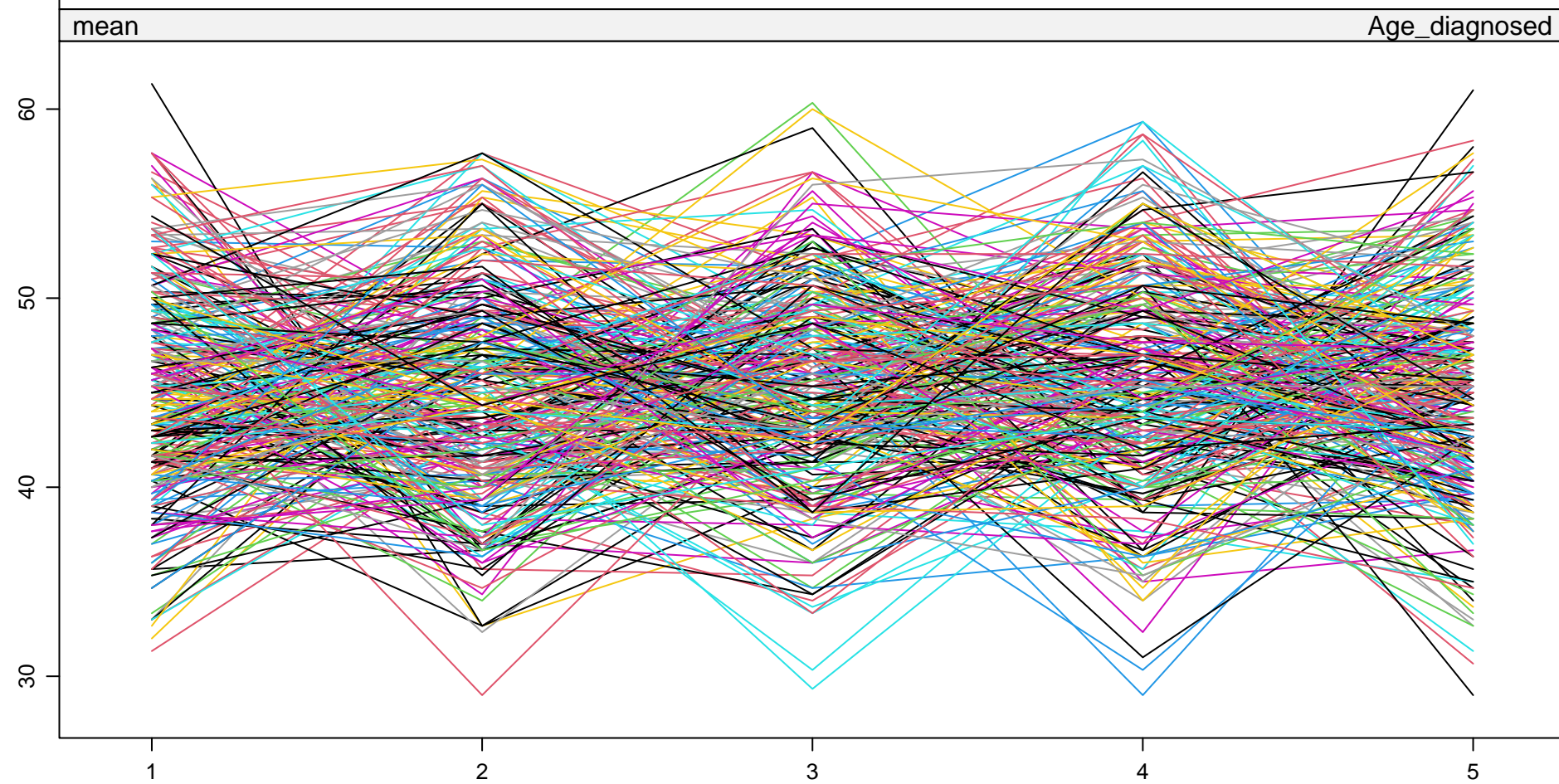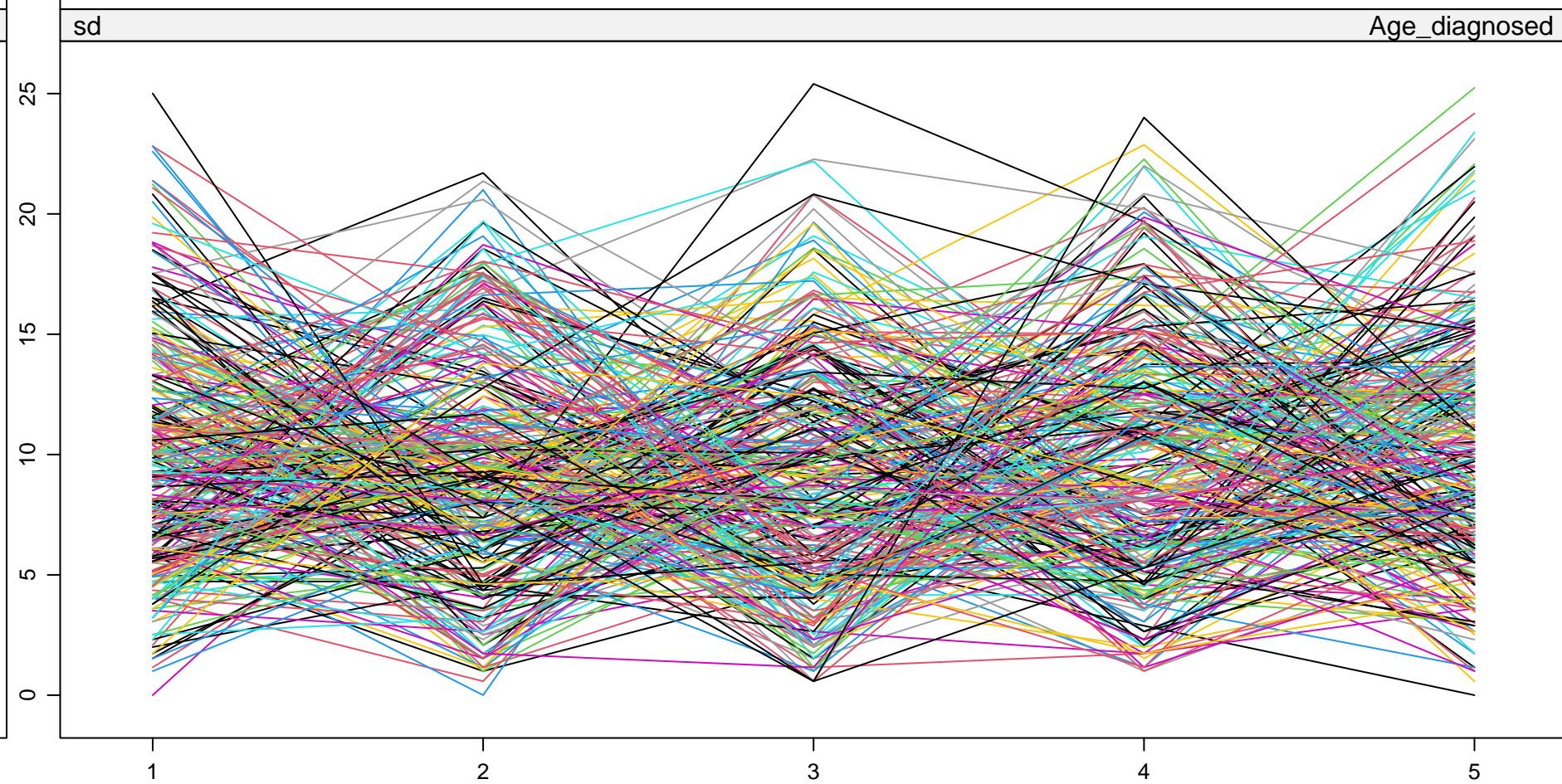

Iteration

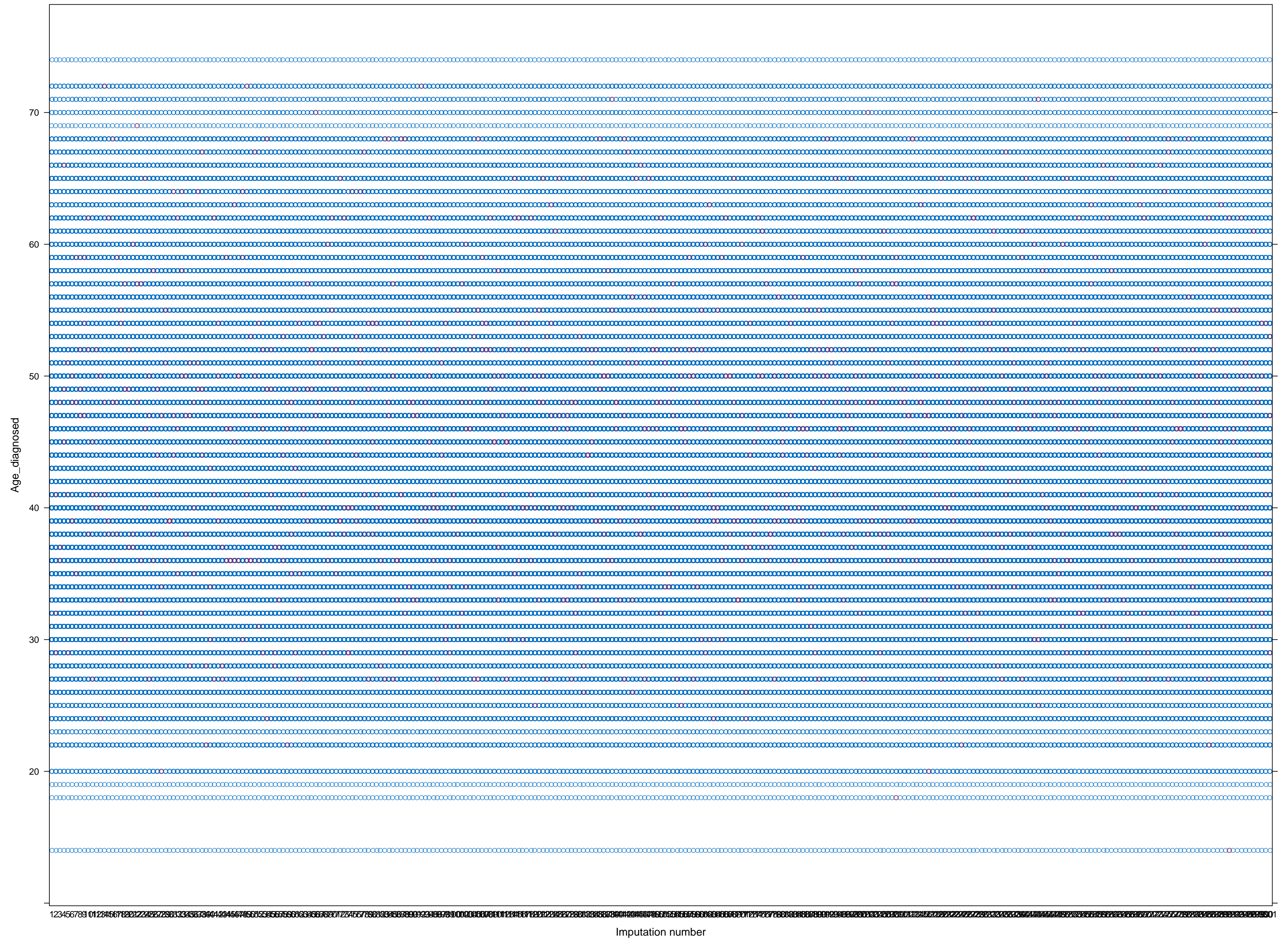

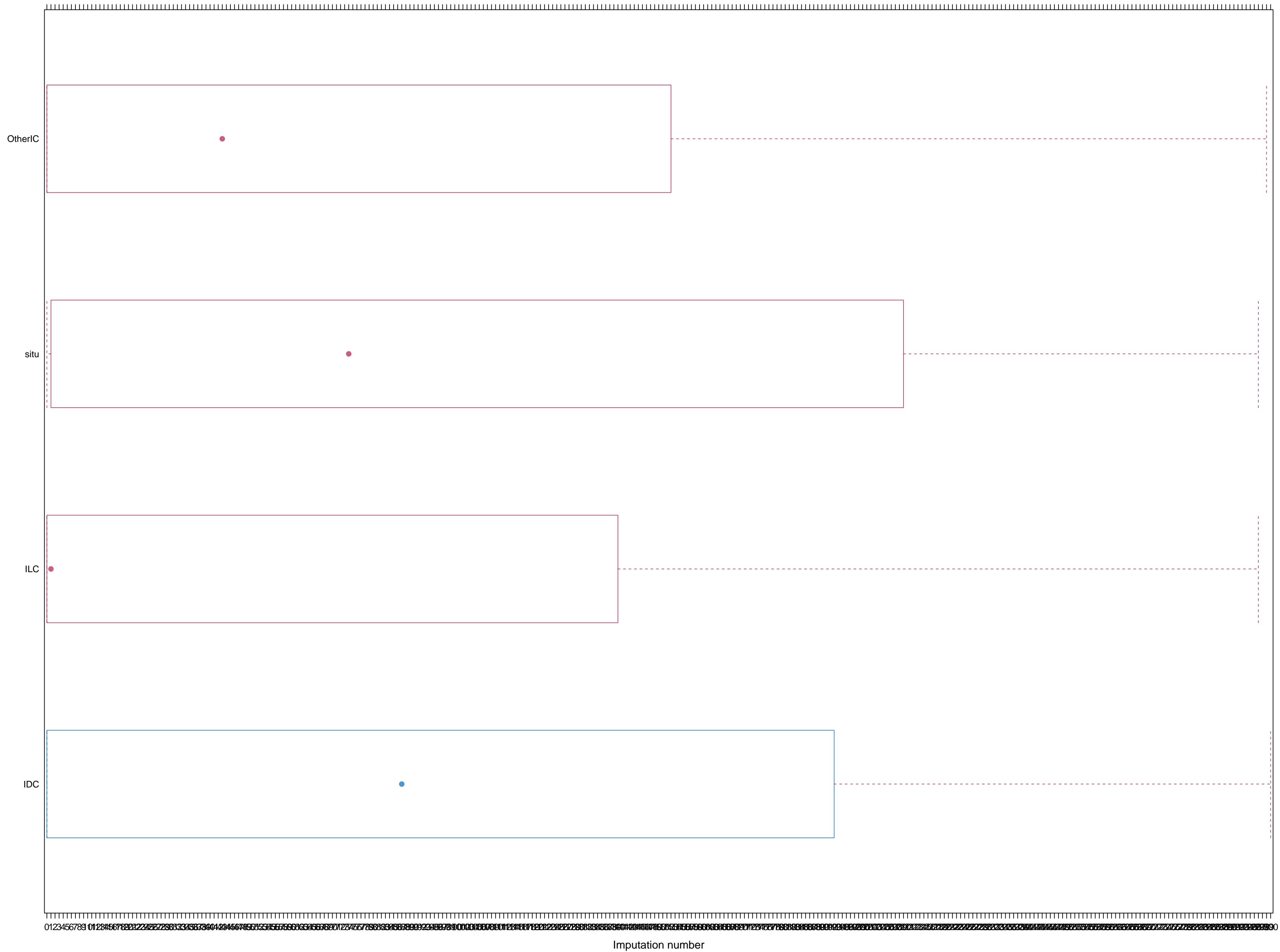

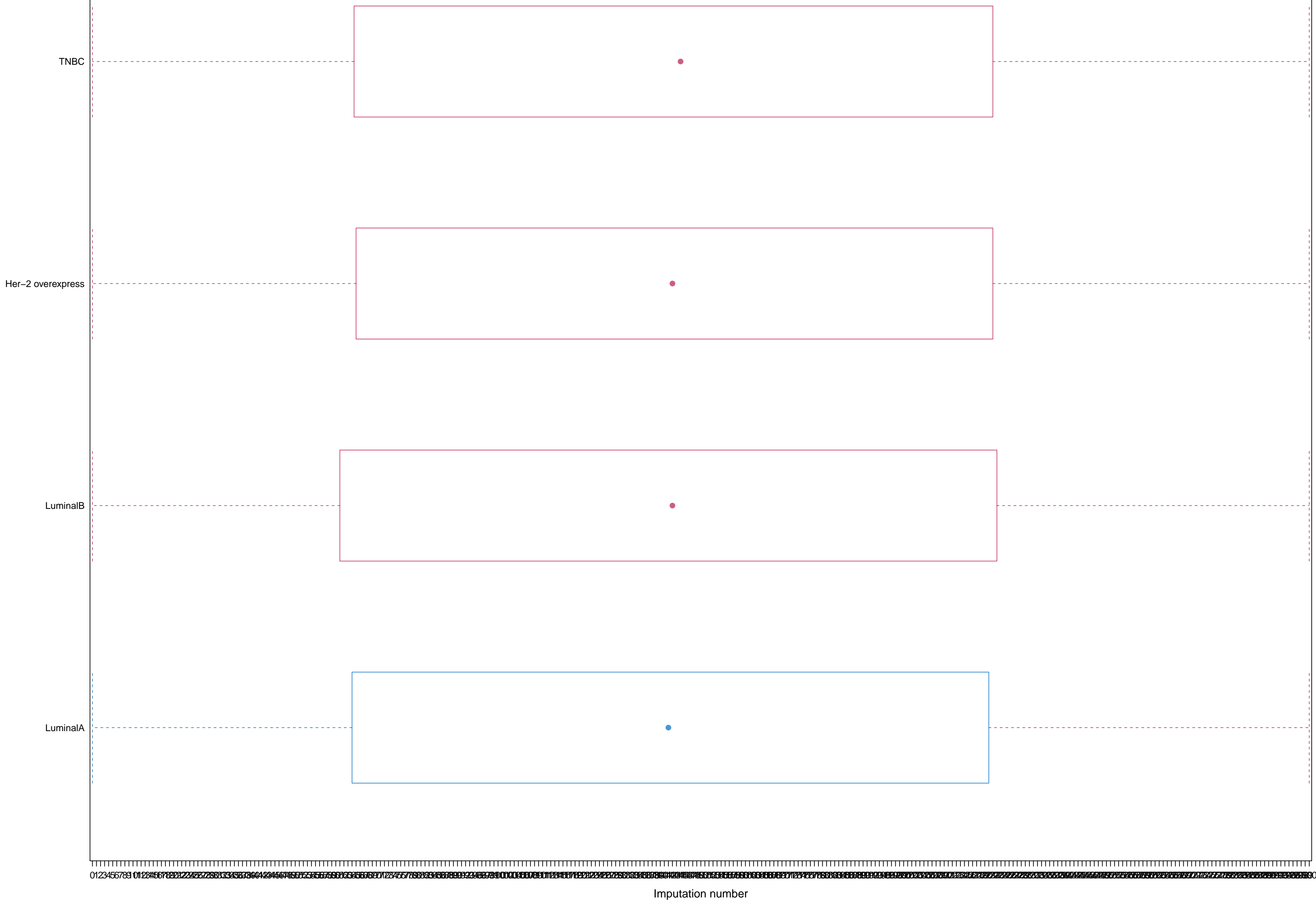

Supplement: Supplementary file 2 [file DataSheet2.pdf]

# Sensitivity Analyses: IDC vs Non-IDC

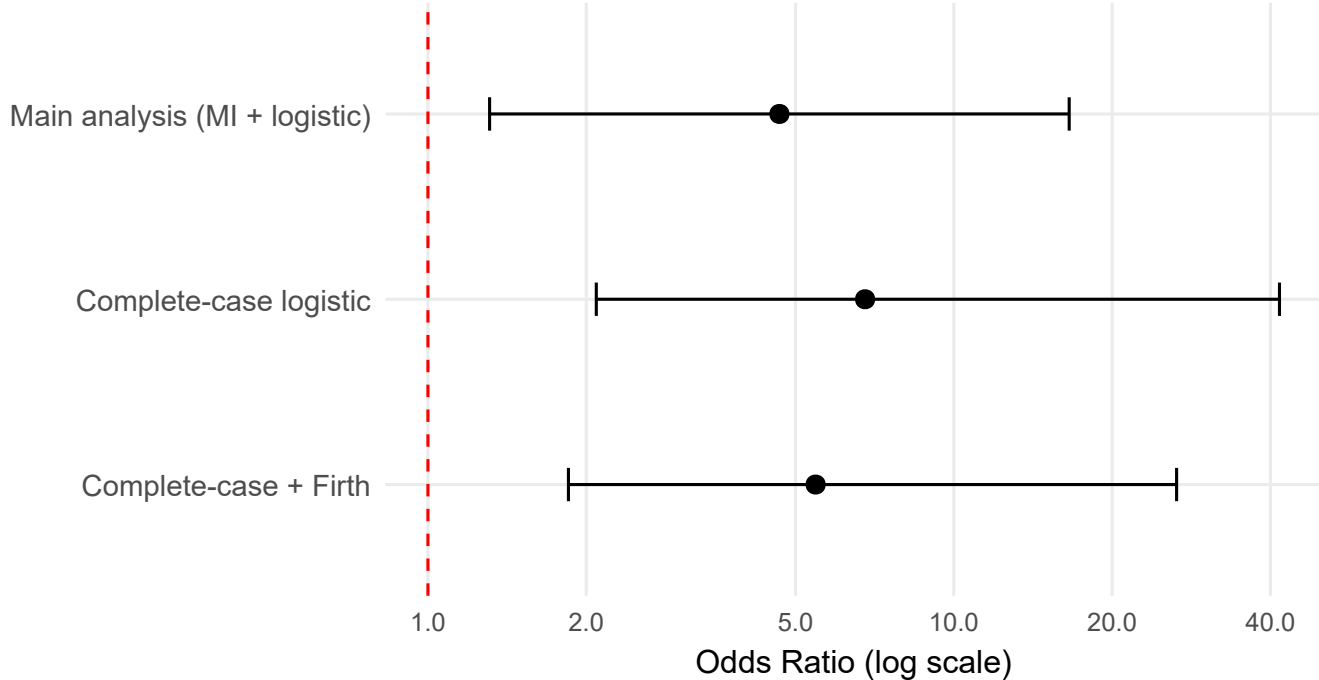

Supplement: Supplementary file 3 [file DataSheet3.pdf]

**ROC of Final Model in TNBC and non-TNBC**

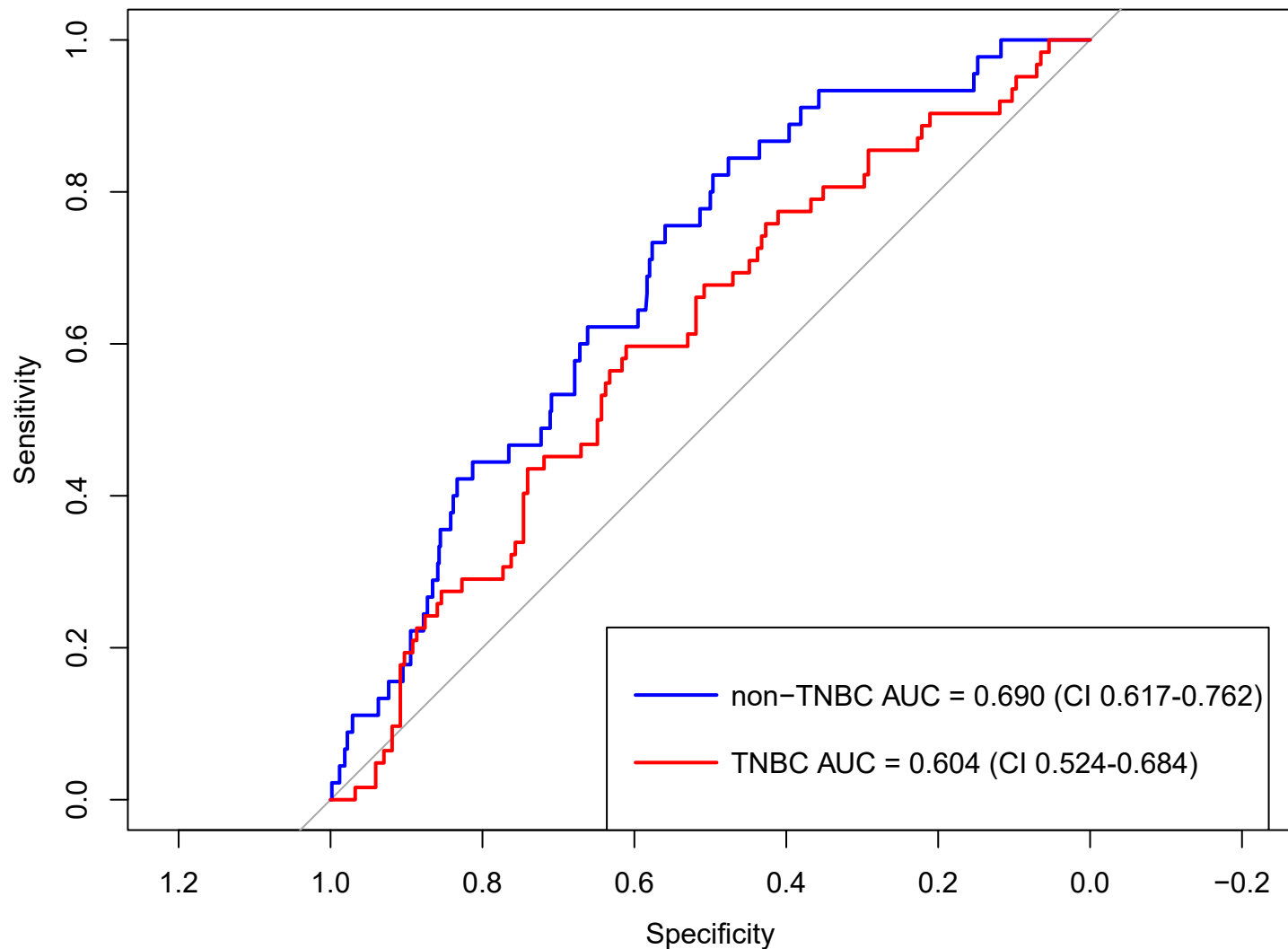

Supplement: Supplementary file 4 [file DataSheet4.pdf]
