# Supplementary material for: Clinical predictors of BRCA1/2 P/LP variants for high-risk breast cancer patients in China: HBRCA-risk prediction
Source: Front Oncol. 2026 Jun 2;16:1779548. doi: 10.3389/fonc.2026.1779548 (PMC13268984; doi:10.3389/fonc.2026.1779548)
Supplement: Supplementary file 5 [file DataSheet5.pdf]

Table S1. List of genes of 98 gene panel.

ABRAXAS1, AIP, ALK, APC, ATM, AXIN2, BAP1, BARD1, BLM, BMPR1A, BRCA1, BRCA2, BRIP1, CASR, CDC73, CDH1, CDK4, CDKN1B, CDKN1C, CDKN2A, CEBPA, CHEK2, DICER1, DIS3L2, EPCAM, FH, FLCN, GATA2, GPC3, HRAS, KIT, MAX, MEN1, MET, MLH1, MRE11, MSH2, MSH6, MUTYH, NBN, NF1, NF2, PALB2, PAX5, PDGFRA, PHOX2B, PMS1, PMS2, POLD1, POLE, PRF1, PRKAR1A, PTCH1, PTEN, RAD50, RAD51C, RAD51D, RB1, RECQL4, RET, RUNX1, SDHA, SDHAF2, SDHB, SDHC, SDHD, SMAD4, SMARCA4, SMARCB1, SMARCE1, STK11, SUFU, TERC, TERT, TMEM127, TP53, TSC1, TSC2, VHL, WRN, WT1, XRCC2，MCPH1，RECQL，FANCC，FANCM，FANCA, FANCB，FANCD2，FANCE，FANCF，FANCG/XRCC9，FANCI，FANCL，FANCP/SLX4, FANCQ/ERCC4，FANCR/RAD51，FANCT/UBE2T

Table S2. Sequencing coverage and quality statistics for each sample.

Sample ID	Total number of sequenced reads	Total number of uniquely mapped non duplicate readsa,b	Total number of covered targeted bases	Median coverage (and range) per targeted base	Percentage of targeted bases with coverage ≥200
1	15666477	11528129	1048863	513 (0–2277)	91.61
2	7373619	4618542	1048591	648 (0–3348)	91.78
3	12505645	8801296	1049476	1150 (0–5103)	98.90
4	12970595	8830083	1050081	1113 (0–5200)	97.07
5	15600704	10512602	1050431	892 (0–3497)	98.23
6	17095322	11868598	1050314	895 (0–4240)	98.02
7	14949525	10310628	1048792	804 (0–3316)	94.70
8	11514818	8022654	1051158	1069 (0–5200)	98.55
9	14424952	9875887	1051373	850 (0–3531)	95.93
10	13773536	9260433	1050629	816 (0–4045)	95.75
11	11222334	7946306	1049655	718 (0–3710)	93.46
12	8241052	5697918	1049641	611 (0–2095)	95.10
13	9291430	5923607	1050629	655 (0–3184)	94.03
14	8719313	6355406	1048975	919 (0–4832)	95.96
15	10087955	7178307	1051034	687 (0–3549)	94.02
16	12289083	8301863	1049295	1150 (0–5200)	97.53
17	8268950	5486523	1049241	802 (0–4293)	94.08
18	10299041	6945291	1050169	1150 (0–4646)	98.37
19	12308512	7925967	1049564	766 (0–3678)	93.10
20	13740081	9149196	1051077	594(0-4633)	90.73
21	8989246	6318494	1050213	772 (0–4846)	91.86
22	13410313	8391790	1050935	1150 (0–5101)	98.47
23	16951456	11367270	1050871	500 (0–2740)	88.89
24	17682479	12084430	1050690	511 (0–2242)	89.63
25	11605267	7927640	1051130	1150 (0–5200)	99.68
26	11656381	7514075	1049272	789 (0–3608)	97.29
27	7654544	4933625	1050621	500 (0–2253)	90.07
28	14280980	10177697	1050490	1150 (0–4143)	98.25
29	8294375	5335474	1049951	1035 (0–5200)	97.99
30	12981506	9220238	1050249	982 (0–4131)	99.17

31	9480724	6309697	1051105	525 (0–2579)	88.68
32	15943151	11357803	1050351	1011 (0–5200)	97.42
33	13822392	9224617	1048644	500 (0–1960)	93.36
34	10889085	7023524	1049495	1150 (0–5103)	97.55
35	14625904	10321451	1050960	801 (0–4189)	94.83
36	14726953	10006644	1049079	500 (0–2455)	89.43
37	15654947	11325686	1049205	841 (0–2523)	99.80
38	14080316	9951489	1049056	500 (0–2720)	89.73
39	16707356	11246009	1050044	633 (0–3005)	92.37
40	17211541	11471664	1050533	1134 (0–5070)	99.35
41	17366582	11821453	1051097	1150 (0–4539)	97.97
42	14304603	9808756	1048768	932 (0–3470)	96.21
43	7592162	4961839	1050046	895 (0–3515)	96.81
44	10200993	7194334	1049656	1035 (0–5200)	97.11
45	15890024	10615793	1049832	1101 (0–4915)	98.70
46	8593219	5734334	1050859	714 (0–3931)	90.27
47	16711722	11092896	1049924	1113 (0–5025)	99.30
48	7461376	5125447	1050840	745 (0–3163)	98.81
49	9741067	6421686	1049213	775 (0–3508)	97.27
50	12047714	8241228	1050536	860 (0–4994)	94.91
51	15725306	11793980	1049703	564 (0–2641)	92.50
52	13432138	9112251	1050103	548 (0–2513)	91.77
53	14466774	9649047	1048718	500 (0–2852)	88.99
54	7210637	4542480	1048357	1132 (0–4450)	99.80
55	9448363	6439122	1050522	581 (0–1897)	95.63
56	8626523	5578971	1049361	580 (0–2957)	90.24
57	15577004	10416861	1050820	534 (0–1817)	93.31
58	7191668	4923044	1050172	913 (0–3369)	99.80
59	14888454	9350996	1050828	703 (0–3535)	94.09
60	15694938	10746699	1051003	905 (0–2902)	99.73
61	17037968	11741468	1049219	543 (0–2182)	91.96
62	14594374	9356263	1050718	1150 (0–5200)	99.80
63	10776270	7201035	1050471	500 (0–2394)	90.29
64	12218559	7637577	1050045	591 (0–2534)	94.00
65	10280116	7476369	1049217	1003 (0–4377)	98.10
66	8955783	6178746	1049965	500 (0–2379)	91.58

67	12088613	8155128	1050039	811 (0–3221)	95.96
68	17166967	11589367	1051050	781(0-3674)	97.75
69	13627128	9728997	1050787	626 (0–2979)	92.81
70	8818380	5971587	1048861	807 (0–4231)	95.48
71	16114130	11117533	1050304	800 (0–4548)	97.48
72	10665361	7738347	1050296	957 (0–4430)	97.55
73	13432257	8994441	1049489	599 (0–2885)	92.46
74	13240307	8569404	1051022	582 (0–2187)	93.69
75	16016236	10494879	1050797	500 (0–3109)	89.23
76	15353072	11098120	1048875	536 (0–2247)	91.18
77	11089819	7571495	1050727	571 (0–2263)	94.88
78	14610710	9715100	1050944	500 (0–2512)	87.90
79	13928899	9878505	1051108	1068 (0–4599)	97.39
80	16370691	11622187	1051078	590 (0–2766)	94.86
81	8962123	5966359	1051267	1033 (0–3992)	99.80
82	16357973	10646454	1050369	500 (0–1909)	90.51
83	12776873	8086280	1050856	1150 (0–3998)	99.80
84	11790155	7382799	1050843	500 (0–2253)	90.21
85	11223731	7542913	1049670	1150 (0–5200)	99.80
86	17731782	11582473	1049488	500 (0–2415)	92.31
87	11998231	8827749	1050397	1150 (0–5200)	99.68
88	17593294	11994336	1050632	764 (0–4103)	93.40
89	15435686	9596914	1049158	616 (0–2996)	94.19
90	9079187	6560274	1050256	811 (0–3293)	96.59
91	13351737	9143955	1050496	980 (0–4827)	97.09
92	9786502	6861800	1050775	500 (0–2504)	90.09
93	16142710	11307339	1049033	957 (0–4682)	98.42
94	16210395	11167288	1048597	504 (0–2396)	89.94
95	12843750	8935400	1050159	538 (0–2612)	89.87
96	15428945	10602197	1050536	500 (0–2608)	90.31
97	12779389	8265816	1050960	687 (0–2269)	97.69
98	17798958	12299115	1050785	1041 (0–4730)	98.78
99	16659235	11840004	1050927	538 (0–2332)	91.49
100	11673140	7864175	1050706	816 (0–3887)	92.69
101	10264611	7149703	1049202	1150 (0–5021)	95.87
102	16029825	11102059	1049482	559 (0–2435)	91.78

103	9165551	6731818	1049462	583 (0–3322)	92.83
104	9083309	6107776	1050622	537 (0–2734)	89.17
105	9928656	6664510	1049801	791 (0–4119)	96.22
106	12539461	8482846	1050157	500 (0–1800)	92.16
107	16009512	10837291	1050114	772 (0–4846)	94.24
108	10121423	6192504	1050077	1150 (0–4452)	99.80
109	16096507	10635312	1050401	782 (0–2828)	99.50
110	7932136	5465214	1050454	775 (0–2325)	97.02
111	17136877	11648832	1049153	500 (0–2088)	88.74
112	9422528	6181365	1050071	1008 (0–4733)	98.54
113	16585619	10947448	1050521	972 (0–3807)	99.71
114	14843677	9935342	1050640	1046 (0–4989)	97.12
115	8951206	5871569	1049897	885 (0–3642)	96.69
116	15284220	10158022	1048834	821(0-4675)	96.71
117	16961793	11259549	1050796	540 (0–2565)	89.12
118	9674548	6838562	1050683	745 (0–3163)	96.37
119	10576574	7514568	1050176	1041 (0–5200)	97.85
120	7346060	5201066	1049663	800 (0–4548)	97.51
121	12448003	8623990	1048321	500 (0–2512)	89.26
122	16823027	11082175	1049080	558 (0–2377)	91.52
123	14876231	10544197	1049022	653 (0–3230)	92.00
124	15725478	10545073	1050189	807 (0–3497)	96.24
125	7980934	5432554	1050029	806 (0–3959)	95.50
126	8916045	6420860	1050254	1150 (0–5200)	98.05
127	12599644	8599506	1050585	1104 (0–4662)	97.39
128	10560812	7259698	1050065	970 (0–4135)	98.61
129	7249764	4622570	1049430	540 (0–2565)	92.23
130	8269367	5743987	1049756	1026 (0–4997)	96.48
131	7219632	4697091	1050884	1093 (0–4681)	96.43
132	8884584	6311237	1050980	694 (0–3836)	96.15
133	11505048	7759718	1049686	500 (0–2394)	88.25
134	9910970	6074582	1050180	718 (0–4283)	93.67
135	17344506	11607974	1048991	762 (0–2668)	98.29
136	13053357	8786984	1050662	947 (0–4306)	97.64
137	15933453	11066807	1049755	831 (0–4101)	93.91
138	12422286	8697033	1050016	935 (0–3829)	97.63

139	8888646	5956878	1049608	710 (0–3556)	94.85
140	15444993	10971908	1049288	784 (0–2699)	98.96
141	15541406	9324844	1051252	640 (0–2547)	95.36
142	15557766	10246039	1048562	758 (0–2767)	99.80
143	14164456	9159149	1050855	733 (0–3729)	95.39
144	13047690	8381503	1050339	628 (0–2895)	94.18
145	14576652	10331803	1050229	1002 (0–4528)	99.80
146	10108505	7125234	1050712	879 (0–4999)	92.23
147	17634730	11581202	1050470	905 (0–3752)	97.89
148	13533755	9681259	1049902	816 (0–4045)	94.21
149	10838087	7402890	1050128	1116 (0–4991)	99.68
150	15698637	10476477	1048679	568 (0–2121)	92.75
151	16209260	10685344	1050713	1150 (0–5200)	97.59
152	8639163	5672922	1050729	711 (0–3367)	94.66
153	9288897	6448197	1051385	500 (0–2094)	90.43
154	12947049	9056521	1049666	733 (0–3729)	93.77
155	7101816	4880049	1050591	1150 (0–5200)	99.80
156	11241879	6953029	1049508	706 (0–3248)	94.81
157	8479231	5841264	1050359	840 (0–3624)	95.89
158	15145411	10555774	1049600	629 (0–3327)	91.08
159	13823953	9122756	1049391	502 (0–1941)	90.06
160	13992984	9533385	1050454	500 (0–2633)	89.92
161	8831662	5511694	1050503	1041 (0–4367)	99.80
162	17767575	11426344	1050006	567 (0–2406)	93.85
163	15597701	10454717	1050273	589 (0–2266)	93.10
164	9599521	6438470	1050848	906 (0–4465)	95.90
165	15220005	10510180	1049341	512 (0–2296)	92.11
166	7275586	4688331	1049479	1150 (0–5200)	99.53
167	13808109	9248172	1049088	716 (0–3739)	94.21
168	9596793	6384810	1050118	1150 (0–5200)	98.90
169	15725411	10942375	1051111	870 (0–3477)	96.22
170	9056878	6544783	1050589	527 (0–2321)	91.52
171	11599734	8486686	1051141	1150 (0–4533)	96.98
172	11213310	7097429	1049121	858 (0–4310)	95.66
173	8492619	5762748	1051032	865 (0–3817)	98.96
174	14984538	9689364	1050677	659 (0–4046)	91.17

175	15562780	10552462	1050338	689 (0–2456)	97.35
176	16797624	11635505	1049530	627 (0–2823)	95.20
177	9058699	6182061	1050583	1150 (0–4101)	99.42
178	9595373	6510745	1050917	1141 (0–5200)	97.49
179	15698490	11373417	1048568	1086 (0–5200)	97.84
180	10920314	7662016	1048588	1004 (0–5200)	98.08
181	14553465	9777314	1050502	719 (0–3757)	94.17
182	15531451	10572668	1049427	847 (0–3566)	97.49
183	12329155	8705948	1049915	1150 (0–5200)	99.80
184	13214997	9227949	1050337	623 (0–3410)	92.55
185	13089659	8734045	1050007	1148 (0–5200)	99.32
186	7970230	4995284	1049012	1011 (0–5200)	96.88
187	13089136	8435428	1051286	1150 (0–5200)	99.14
188	11990264	8205889	1050720	1150 (0–5200)	99.07
189	10816407	6775443	1050172	1150 (0–5200)	99.80
190	7811863	5276352	1050797	1132 (0–4450)	99.52
191	7891780	5259026	1049563	1150 (0–5200)	97.70
192	10988592	7801654	1049846	1150 (0–5200)	97.34
193	14743800	9442795	1049719	1014 (0–3754)	99.37
194	12993666	8082670	1050850	567 (0–2909)	90.91
195	8635301	5763293	1050214	518 (0–2319)	91.25
196	11958881	8359771	1049948	984 (0–4405)	99.33
197	12220988	8702536	1049360	938 (0–4510)	97.57
198	13943951	9101498	1049903	1122 (0–4249)	98.80
199	14318779	9938778	1050100	1038 (0–4351)	97.25
200	14156034	9154100	1050033	500 (0–1800)	92.31
201	15317962	10072099	1050986	871 (0–3227)	96.03
202	15054704	10493548	1050756	1099 (0–5200)	96.74
203	17502875	12279046	1049766	1054 (0–5200)	98.24
204	12928631	8403850	1049728	1150 (0–4770)	99.21
205	14654693	10173976	1049409	1028 (0–4666)	99.42
206	8830193	5509555	1050620	733 (0–3933)	95.42
207	12149527	8187812	1051380	1150 (0–5200)	97.67
208	15663705	10118324	1050854	1062 (0–5200)	98.89
209	10478714	6871616	1050603	827 (0–3645)	95.82
210	11887853	7628645	1050252	744 (0–3473)	94.42

211	17177546	11966030	1049493	691 (0–3195)	93.90
212	11289672	7864569	1049759	1150 (0–5200)	97.60
213	11342517	7477713	1051167	781 (0–2720)	98.14
214	13047001	9018967	1050637	745 (0–3155)	97.93
215	10068259	6925333	1050923	708 (0–4116)	93.06
216	11959811	8086584	1050954	559 (0–1913)	91.54
217	8229254	6128697	1051154	902 (0–5200)	96.00
218	17482913	11447351	1048361	1007 (0–4525)	97.53
219	9748071	6632872	1050531	580 (0–2580)	93.03
220	15653778	10464995	1049335	500 (0–3109)	86.42
221	17066140	11414630	1051017	823 (0–5017)	95.01
222	10856806	7434549	1050909	589 (0–2905)	92.52
223	11848517	7952347	1051039	887 (0–4218)	96.72
224	14710888	9623675	1050497	969 (0–5200)	94.84
225	15300009	10461027	1050382	500 (0–2763)	87.22
226	10079492	7211574	1050842	1150 (0–4273)	99.80
227	10372538	7346692	1049951	762 (0–3113)	97.74
228	14060885	9647051	1050246	649 (0–2433)	98.17
229	11397874	7499096	1050221	656 (0–2607)	93.67
230	8071012	5533428	1049605	734 (0–3610)	92.41
231	7060460	5030855	1050957	731 (0–2746)	95.93
232	12117923	8673230	1049435	742 (0–3049)	98.15
233	13456428	9295574	1049295	655 (0–3446)	92.65
234	11388712	7619837	1050770	941 (0–3793)	98.64
235	14287797	9628795	1050805	796 (0–2388)	98.56
236	11042487	7764650	1050788	572 (0–3577)	91.38
237	17444750	12221624	1048393	949 (0–3968)	97.43
238	15224279	9549718	1050507	500 (0–2291)	91.25
239	11938357	8353967	1050857	1150 (0–5200)	99.65
240	16202256	11363268	1050731	1150 (0–5103)	99.60
241	16366256	10693454	1050614	860 (0–4994)	95.70
242	8089457	5315182	1048134	906 (0–3121)	99.80
243	13161023	8650544	1050206	762 (0–2315)	96.82
244	10485268	6916000	1050140	500 (0–2331)	91.93
245	14505643	9247311	1050090	803 (0–4843)	92.51
246	16640687	11471969	1048191	1150 (0–4450)	99.38

247	17786010	11675242	1050485	572 (0–3272)	92.91
248	7619381	5165091	1049946	1004 (0–4908)	97.46
249	15763908	10484149	1051019	738 (0–2856)	95.09
250	16715073	11494209	1050065	750 (0–3460)	96.01
251	7341858	4769869	1051203	500 (0–1800)	92.36
252	8783250	6037237	1047602	1086 (0–4609)	99.80
253	10458663	7234188	1050163	500 (0–1845)	91.49
254	15486746	10915490	1050016	688 (0–3132)	96.59
255	17942823	13260340	1049980	681 (0–3771)	92.23
256	14210554	8771279	1050970	1150 (0–5200)	98.23
257	11071334	7634896	1049621	1150 (0–4045)	98.11
258	14666433	9661148	1048673	770 (0–3873)	93.62
259	10669428	6639033	1050250	1103 (0–5182)	97.98
260	8102737	5714447	1049819	691 (0-2672)	97.28
261	9837270	7082500	1050765	820 (0–3344)	97.89
262	10667454	7128539	1050700	868 (0–4482)	97.79
263	9861017	6767887	1050484	1010 (0–5200)	94.27
264	8581386	5818846	1050255	637 (0–3020)	94.22
265	11385664	7890560	1049968	500 (0–1800)	88.90
266	15933155	11255437	1051185	710 (0–3556)	94.47
267	12041235	8312644	1050530	887 (0–4218)	99.25
268	17876759	12341633	1049522	1150 (0–5200)	98.17
269	11607477	8331755	1050422	1122 (0–4249)	99.80
270	9642806	6509474	1051067	706 (0–3248)	96.69
271	10501390	7431749	1050420	1027 (0–4601)	99.14
272	11344861	7434741	1050532	696 (0–3275)	93.53
273	16543973	12047529	1049713	795 (0–4414)	97.51
274	15878441	11148805	1050266	821 (0–4487)	93.70
275	9326179	6228836	1051249	734 (0–3610)	95.51
276	7371277	4544861	1049398	1150 (0–5056)	99.45
277	8554457	5816951	1049251	500 (0–1800)	92.78
278	13068701	8423375	1050666	1150 (0–5200)	99.80
279	9394554	6472745	1049723	1024 (0–3589)	99.68
280	15230301	11019657	1050438	782 (0–3595)	95.40
281	15338001	10503491	1049588	725 (0–3966)	93.41
282	12960085	8743646	1050698	1150 (0–4691)	99.80

283	11322448	7815503	1050986	967 (0–3456)	98.69
284	14657911	10092991	1051009	782 (0–2828)	99.15
285	13726096	8360758	1048203	500 (0–2424)	87.42
286	9018316	6197640	1050514	890 (0–3639)	99.80
287	10235216	7150407	1050115	1009 (0–5200)	96.57
288	7440412	5001592	1050201	695 (0–3175)	93.60
289	16555494	11743752	1050343	500 (0–2068)	94.32
290	9008418	6091220	1048726	838 (0–3869)	94.22
291	8698410	6115069	1050245	683 (0–3793)	94.01
292	8841317	6615013	1050225	868 (0–4482)	93.75
293	17657806	11956246	1050003	1010 (0–5200)	96.31
294	13238833	8231060	1049498	1149 (0–5200)	99.00
295	7963200	5334566	1050700	500 (0–1996)	89.64
296	16519078	10377670	1050056	706 (0–3248)	94.12
297	12020591	8394425	1050942	739 (0–2682)	97.14
298	8995197	6470070	1050963	640 (0–3619)	92.39
299	7926588	5555558	1050674	1150 (0–5200)	99.80
300	14643497	10098291	1050064	741 (0–3249)	94.37
301	10875921	7185202	1049104	500 (0–2373)	92.19
302	15038622	10937131	1051008	500 (0–2415)	88.07
303	8920006	6267181	1050379	713 (0–2929)	94.28
304	8091546	5571305	1049904	649 (0–2433)	96.18
305	13123079	9004346	1050304	849 (0–4144)	95.57
306	12006157	8204393	1049424	1107 (0–5200)	94.98
307	7542961	5231545	1048873	782 (0–3595)	96.36
308	7487246	4953563	1050482	1150 (0–5200)	99.80
309	7300998	5298754	1049611	741 (0–3249)	95.05
310	8899744	6092499	1049930	500 (0–2894)	88.14
311	10701299	7469524	1049902	1150 (0–5200)	99.25
312	12623649	8419885	1049539	1150 (0–4388)	99.04
313	14040128	9705204	1050930	917 (0–3130)	97.38
314	7083048	4331620	1048729	1150 (0–5200)	99.26
315	9295422	6009035	1050758	862 (0–3225)	97.47
316	7203085	4891310	1050566	615 (0–2654)	92.18
317	11262494	7211666	1049091	756 (0–3033)	97.96
318	7796837	5847628	1049863	801 (0–4318)	98.54

319	16122643	10156581	1050866	1023 (0–4132)	99.80
320	11171522	7712562	1051027	1150 (0–5200)	98.67
321	13354914	9744723	1049957	500 (0–2615)	89.17
322	15877549	11025983	1049939	655 (0–3184)	93.90
323	15533518	10418224	1050208	895 (0–3515)	97.00
324	11477442	7614545	1049356	733 (0–3381)	97.86
325	15794971	11059602	1050726	645 (0–3114)	93.89
326	15645594	10263383	1050253	566 (0–2441)	91.87
327	15466798	10461981	1050230	704 (0–3514)	93.80
328	12227168	8568016	1049755	637 (0–3020)	94.75
329	15916946	10676508	1049064	500 (0–2319)	89.05
330	15424375	10529069	1049784	829 (0–2876)	97.11
331	10253536	6878866	1050495	642 (0–3582)	93.15
332	8830302	5747697	1050357	1150 (0–3462)	98.99
333	8561347	6127138	1048782	825 (0–3590)	97.36
334	13678351	9872924	1050340	764 (0–4103)	93.12
335	16933680	11354016	1050449	555 (0–2854)	93.84
336	7093290	4766923	1051044	789 (0–3608)	98.49
337	16357019	11035476	1050056	1103 (0–5182)	99.22
338	12619534	8785267	1050479	1150 (0–5200)	97.71
339	14751791	9630574	1050672	700 (0–3323)	94.20
340	12501086	8467539	1049904	575 (0–2273)	94.47
341	9034363	6214637	1051019	1150 (0–5200)	99.80
342	15895316	11114979	1048321	987 (0–4021)	97.67
343	13052639	7831583	1050293	801 (0–4189)	93.99
344	11476998	7943254	1049695	804 (0–3316)	96.69
345	12826272	8854943	1049138	816 (0–3251)	97.71
346	10632519	7551554	1050958	1150 (0–5200)	97.40
347	15965309	10604351	1050901	514 (0–1860)	91.16
348	10936504	7266754	1049754	949 (0–4455)	96.09
349	16686239	11948607	1051158	500 (0–2173)	89.76
350	13215130	8366569	1050548	500 (0–2412)	90.11
351	15637313	11397611	1048919	546 (0–2502)	93.76
352	10527815	7895861	1050245	1150 (0–4790)	99.42
353	13999807	9720396	1050230	590 (0–2766)	91.58
354	17490736	12320876	1049913	540 (0–1919)	91.18

355	15747097	10004328	1050312	646 (0–3203)	92.75
356	15428830	10170117	1050577	500 (0–2220)	88.44
357	17470611	12434461	1048377	502 (0–1941)	92.60
358	7635717	5275169	1050179	855 (0–3957)	98.36
359	15325518	10337938	1048827	737 (0–3914)	94.39
360	14986872	10295772	1050936	820 (0–3697)	94.72
361	15871168	11033784	1049434	1150 (0–3462)	99.80
362	17795804	11944671	1049667	1150 (0–5200)	99.80
363	7789057	5289795	1051006	801 (0–4318)	93.46
364	16206792	11002966	1050469	653 (0–3230)	94.46
365	10756840	6975075	1051112	756 (0–4105)	94.01
366	9120118	6290217	1049903	850 (0–3484)	98.68
367	10315448	7572649	1049667	500 (0–2800)	89.94
368	15409695	11279263	1049315	785 (0–3129)	94.07
369	8880031	5645107	1051307	824 (0–3195)	98.29
370	7262831	5063057	1049891	767 (0–2914)	98.58
371	16861836	11807755	1049570	520 (0–2529)	88.89
372	10090306	6737143	1051071	952 (0–3498)	99.80
373	9530527	6205078	1049699	500 (0–1800)	90.19
374	17410528	11149277	1050459	1150 (0–4450)	98.97
375	13141225	8760714	1049120	1045 (0–5172)	96.67
376	13350057	9218218	1050309	863 (0–3252)	98.79
377	13767692	9296748	1050792	769 (0–4364)	93.48
378	7540458	4989310	1050316	846 (0–3834)	99.62
379	14667203	9424049	1048978	1132 (0–5200)	99.57
380	7691045	5310832	1050654	500 (0–2574)	91.40
381	9099903	6479097	1049932	1150 (0–5200)	97.99
382	7327090	4778672	1050839	608 (0–2129)	96.08
383	8460954	5867273	1050473	657 (0–2281)	95.39
384	15102119	9417919	1050368	562 (0–2753)	94.66
385	17862035	11793873	1050933	738 (0–2856)	96.88
386	11433297	8024895	1050673	936 (0–3790)	96.77
387	9941843	6427073	1050387	856 (0–3389)	97.20
388	14500445	9597365	1050784	908 (0–4374)	99.80
389	16899209	12210338	1050258	500 (0–1800)	93.00
390	13896819	9144311	1050931	941 (0–4202)	98.46

391	10296305	6737713	1050838	500 (0–1800)	91.29
392	8668473	6044313	1050022	500 (0–2524)	90.44
393	17455549	12893402	1050275	738 (0–3708)	92.20
394	16911816	11378437	1051187	1113 (0–5025)	96.20
395	14762665	10379070	1049180	850 (0–3321)	97.12
396	7534875	5427921	1050301	961 (0–4573)	97.13
397	12065962	8322697	1049664	756 (0–3033)	98.03
398	8267314	5554829	1049706	500 (0–1800)	90.18
399	10854441	7095659	1050250	673 (0–3244)	94.97
400	8774063	5591216	1049568	1061 (0–5200)	96.41
401	14288391	10192639	1050235	844 (0–4104)	95.97
402	14878125	10197894	1049424	582 (0–1800)	97.43
403	7719500	5557917	1049894	779 (0–4927)	94.08
404	10416858	7392508	1048292	1134 (0–4135)	98.89
405	8294062	5486446	1049875	1004 (0–4908)	97.12
406	12885819	8303537	1049334	762 (0–3113)	97.77
407	9826535	6803697	1050911	500 (0–1800)	92.77
408	13229812	8745234	1051232	510 (0–2399)	91.55
409	13684058	9459799	1050658	730 (0–3536)	96.55
410	10885054	7163078	1050842	534 (0–1817)	93.77
411	17650882	11546558	1050128	500 (0–2247)	87.78
412	12786423	8640685	1048184	906 (0–4844)	97.13
413	9411267	6663394	1050162	531 (0–2224)	92.83
414	15211751	10483881	1049958	862 (0–3225)	96.99
415	8805120	6298754	1050627	1150 (0–4579)	97.18
416	16753805	10772582	1049122	1009 (0–4212)	95.53
417	14188679	9198346	1049685	950 (0–3549)	98.21
418	9451480	6401725	1048837	1070 (0–5200)	98.70
419	12625894	9405925	1048962	913 (0–3369)	99.80
420	9271562	6492213	1050822	683 (0–2726)	95.83
421	13487192	8739722	1050627	655 (0–3184)	94.24
422	12595168	8535944	1050218	1150 (0–5200)	97.03
423	13336446	9157090	1049841	806 (0–3959)	96.70
424	14234044	9436309	1051213	1150 (0–5200)	99.47
425	12382578	8647971	1048982	558 (0–2034)	91.88
426	16365103	11051462	1051258	1150 (0–5200)	99.51

427	7606164	5103889	1050536	500 (0–2524)	91.62
428	12220531	8771991	1049513	919 (0–5200)	96.28
429	14604659	9182098	1051071	1089 (0–4443)	99.80
430	15084169	9821994	1050619	634 (0–2704)	93.32
431	16919415	11090351	1048982	657 (0–2281)	93.10
432	15849009	10374279	1050287	887 (0–4054)	97.93
433	12893704	8485829	1051217	829 (0–2876)	99.80
434	12640417	8780720	1049553	678 (0–3213)	94.01
435	15203287	10838362	1050945	688 (0–3132)	95.60
436	12294610	8698206	1051270	1062 (0–3981)	98.96
437	15480879	10053486	1048601	700 (0–3323)	98.28
438	10826107	7326865	1051023	850 (0–3359)	99.80
439	16035220	11121515	1049340	608 (0–2129)	95.74
440	7138771	4905764	1050825	1150 (0–5200)	99.80
441	17433652	12537802	1050203	772 (0–4846)	92.32
442	7723097	5095236	1049938	1045 (0–5172)	98.15
443	11653182	7978812	1050282	521 (0–1950)	88.36
444	14563782	9731564	1050460	588 (0–3385)	89.30
445	11900839	8182211	1048480	804 (0–3165)	96.62
446	10516727	6810980	1048458	702 (0–3109)	90.91
447	14840751	9678127	1050049	761 (0–3588)	96.16
448	14363655	9452674	1050468	874 (0–4329)	94.08
449	8738228	5794231	1049903	608 (0–2594)	93.37
450	15626269	11351511	1050432	500 (0–2350)	90.86
451	11779463	8425403	1050018	589 (0–2905)	93.52
452	8726712	5878492	1049124	955 (0–4863)	94.39
453	9452964	6477057	1049757	608 (0–2129)	95.55
454	14877191	9404868	1049840	961 (0–4573)	99.25
455	14630763	9870223	1049987	733 (0–3729)	93.17
456	15552639	10650267	1050546	893 (0–4878)	93.83
457	9450666	6751277	1050277	937 (0–4134)	99.80
458	16938447	11684211	1049518	512 (0–2296)	91.62
459	15149110	10498132	1049989	531 (0–2224)	93.43
460	9870531	6800133	1051345	1150 (0–5200)	97.00
461	11850209	8153774	1050832	1133 (0–5200)	97.58
462	7905981	5044785	1050262	821(0-4675)	93.72

463	8168674	5452568	1049331	988 (0–4413)	96.19
464	17083391	12352088	1050995	567 (0–2909)	93.03
465	10145996	7150055	1050985	952 (0–5061)	94.29
466	16790314	11666364	1048665	687 (0–2293)	95.86
467	17060314	11635159	1049819	571 (0–2263)	94.88
468	12882792	9015162	1050403	748 (0–4536)	93.70
469	15277801	9955830	1050834	662 (0–3308)	94.05
470	10892124	6890338	1050010	1041 (0–4730)	95.75
471	16070845	10743961	1050897	769 (0–4654)	94.49
472	15892047	11063053	1050290	571 (0–2943)	89.10
473	13896173	9494350	1049157	930 (0–4060)	97.05
474	11830148	8404359	1048447	1150 (0–5200)	99.48
475	14297828	9873272	1048785	696 (0–3275)	96.64
476	9841959	6705772	1050684	500 (0–2740)	88.13
477	11538087	8305398	1049518	1150 (0–4799)	98.37
478	9919913	7082533	1048424	961 (0–4305)	98.97
479	9956219	6740930	1050653	702 (0–3103)	95.32
480	17656910	11422405	1050950	1150 (0–4891)	99.80
481	11914068	7777255	1051255	546 (0–2502)	90.07
482	7120609	4807525	1049498	500 (0–2234)	92.66
483	10194581	6820130	1049386	1150 (0–4403)	99.80
484	7086024	5170905	1047354	855 (0–3957)	97.64
485	7196249	4530729	1050445	721 (0–3197)	96.05
486	17124021	11611324	1050871	513 (0–2748)	90.59
487	15486289	10035320	1050210	1043 (0–5200)	98.41
488	7337664	5015323	1049443	810 (0–4145)	93.35
489	8241376	5698647	1050330	1150 (0–5200)	99.71
490	16524654	11036041	1051160	1150 (0–5200)	96.54
491	16339998	11371241	1049226	500 (0–1800)	91.50
492	9593657	6466520	1050856	840 (0–3624)	98.65
493	15151828	10221698	1050536	757 (0–3715)	97.11
494	8826518	5891377	1049315	703 (0–2223)	95.73
495	12079100	8449629	1050466	869 (0–3343)	97.89
496	8124068	5558122	1048934	554 (0–2742)	91.53
497	7391269	5374910	1051223	765(0-4197)	92.62
498	14388851	10332645	1050173	634 (0–2733)	93.66

499	17406335	12125483	1050461	739 (0–2682)	96.23
500	14798413	9104232	1049992	1035 (0–3886)	99.80
501	9358022	5946744	1049658	1150 (0–3450)	97.73
502	9457243	6710742	1050136	804 (0–3316)	97.72
503	9960770	6631192	1049959	878 (0–4083)	95.84
504	13721941	9427595	1048758	1113 (0–5025)	97.88
505	14724734	9734312	1049335	879 (0–4999)	96.95
506	10755292	7480699	1050186	533 (0–2308)	91.67
507	12111187	8583679	1049923	917 (0–3130)	98.40
508	12064258	7765898	1048985	542 (0–2937)	90.85
509	15326868	10048185	1050361	637 (0–3215)	91.67
510	13452072	8475991	1050837	1071 (0–4622)	97.44
511	8589664	5901118	1050195	545 (0–2353)	91.71
512	16076317	10128748	1049702	596 (0–2909)	93.05
513	16112360	10779452	1050977	691 (0–3711)	93.48
514	13314632	9261503	1050643	748 (0–4259)	92.22
515	7837142	5813144	1050239	1150 (0–3450)	99.19
516	9328212	6446285	1049113	500 (0–2513)	89.96
517	9770335	7011061	1050607	500 (0–2608)	87.63
518	9500972	6534529	1049691	1008 (0–4733)	98.45
519	11504217	7929596	1050570	500 (0–2567)	90.53
520	7854048	5291055	1050503	500 (0–2271)	88.41
521	11854664	7924865	1050169	776 (0–3913)	93.45
522	15600703	11158112	1051090	1102 (0–5200)	97.26
523	7144390	4938490	1049575	1150 (0–5200)	99.80
524	8320952	5742197	1049013	500 (0–2032)	92.37
525	17558180	11619209	1050604	821 (0–4101)	95.89
526	11888516	7961130	1050418	988 (0–4413)	98.15
527	14951497	11094380	1050900	564 (0–2641)	93.13
528	12466832	8452200	1049960	576 (0–3148)	89.87
529	12748507	9176185	1050638	525 (0–2579)	89.34
530	8929902	5932959	1048993	649 (0–2433)	96.79
531	9965293	7016028	1049385	982 (0–4131)	97.25
532	7359957	5068757	1050693	698 (0–3140)	93.68
533	13252910	8851468	1049228	850 (0–3484)	97.33
534	12012639	8402323	1050589	524 (0–2802)	89.29

535	16812338	10653923	1051008	616 (0–2339)	93.58
536	13960803	9734011	1049308	1150 (0–4273)	99.80
537	11351390	7986919	1049273	500 (0–3100)	85.98
538	10102533	6952484	1051251	500 (0–2451)	89.64
539	11260661	8100106	1050245	500 (0–2574)	91.82
540	14761172	10007440	1050703	567 (0–2406)	94.86
541	14381026	9714754	1051101	1150 (0–5200)	99.80
542	9029261	6236070	1049903	616 (0–3070)	94.98
543	7765974	5483020	1051202	730 (0–3536)	96.58
544	14247101	9931586	1049517	1150 (0–5200)	98.48
545	9514455	6405146	1050083	887 (0–3907)	98.95
546	9589001	6234223	1050931	698 (0–3140)	92.08
547	11311876	7917232	1050584	500 (0–2437)	89.86
548	17489468	11478563	1051203	663 (0–2891)	95.63
549	7309673	4791138	1050313	711 (0–3619)	95.07
550	8093277	5344482	1050732	774 (0–4321)	95.67
551	13825670	9425391	1049742	1103 (0–5182)	98.00
552	7113863	4667485	1049297	1018 (0–4442)	99.80
553	11436185	7386968	1050770	629 (0–2740)	93.18
554	9396666	6204005	1050216	806 (0–3651)	97.59
555	9016739	6081791	1049572	574 (0–2941)	93.56
556	13253923	9193299	1049437	906 (0–5200)	96.45
557	7431555	5068454	1049423	521 (0–1950)	91.27
558	17351436	11236549	1050050	799 (0–3174)	99.80
559	9669009	6641254	1049422	944 (0–3926)	96.79
560	15124321	10430982	1050271	772 (0–4846)	94.82
561	15369190	10241277	1050121	1014 (0–3380)	98.39
562	7865764	5160827	1050586	693 (0–3300)	95.57
563	9902137	6496803	1050020	827 (0–3645)	96.34
564	12550318	8333984	1049247	958 (0–5200)	96.98
565	9978170	7447978	1049907	710 (0–3663)	93.49
566	14867113	9713099	1049361	611 (0–3051)	94.26
567	12658131	8708541	1050918	1150 (0–5200)	99.80
568	7442990	4901781	1049921	1150 (0–5200)	99.80
569	7895640	5249183	1049691	626 (0–2979)	93.61
570	8514629	5609573	1050261	706 (0–3830)	95.14

571	8250419	5808593	1049122	1033 (0–3992)	98.80
572	8629277	5966051	1048808	672 (0–2809)	96.75
573	14056851	9504114	1050167	756 (0–4105)	92.97
574	10857015	7546581	1049999	893 (0–3915)	98.36
575	12437423	8734852	1050768	871 (0–3227)	98.97
576	11841328	7749895	1050801	1150 (0–4452)	99.80
577	17250705	12013694	1050296	999 (0–4663)	99.80
578	11546306	7602908	1050826	930 (0–4060)	96.43
579	11487843	8100515	1050531	500 (0–1807)	91.07
580	7550891	5302281	1050903	804 (0–3165)	97.46
581	12149760	8408984	1049843	1150 (0–4491)	98.22
582	17259739	11627924	1050361	687 (0–2293)	96.46
583	7214958	4792296	1050501	718 (0–3710)	95.03
584	12928904	8689402	1050927	855 (0–3254)	94.87
585	7649470	5378148	1050184	500 (0–1800)	92.56
586	9008407	5941315	1050189	500 (0–2068)	89.99
587	13565337	9244643	1050009	782 (0–3595)	94.76
588	7470148	4688049	1050622	1150 (0–5199)	97.56
589	7648076	5260433	1050080	844 (0–4104)	97.24
590	13701397	8891304	1050380	500 (0–2220)	89.12
591	9171425	5873893	1051081	912 (0–4262)	99.80
592	14706520	10229288	1049942	1058 (0–5171)	98.36
593	7753519	5321319	1050295	526 (0–2441)	89.76
594	13189396	8793353	1049923	1140 (0–5054)	96.28
595	9030338	6058789	1050484	829 (0–3327)	97.10
596	10053319	7521200	1050149	1150 (0–4691)	99.80
597	17112241	11536657	1049270	500 (0–2883)	88.31
598	9435890	6359446	1049610	732 (0–2899)	95.89
599	17679526	12368051	1050626	709 (0–2742)	96.00
600	9822933	6705295	1048533	502 (0–1941)	91.15
601	7468780	5300017	1050335	718 (0–4283)	92.20
602	8243338	5674083	1049847	1150 (0–4045)	99.80
603	11311472	7950072	1050516	958 (0–4634)	98.58
604	9822899	6810215	1050863	748 (0–4259)	93.02
605	10408562	7279323	1049579	622 (0–2365)	91.76
606	17785307	12071251	1049953	590 (0–2766)	93.43

607	7014178	4816467	1050488	645 (0–3159)	95.08
608	11907881	8000535	1050276	548 (0–1813)	93.93
609	13020380	9089854	1051080	748 (0–4259)	93.99
610	14735226	9424928	1050183	500 (0–2220)	90.25
611	11740855	8239545	1048800	1093 (0–4681)	98.68
612	13431589	9535633	1050894	949 (0–5200)	95.91
613	14588549	10244850	1051172	1150 (0–5200)	98.53
614	7433997	5031902	1050130	733 (0–4077)	93.93
615	9156764	6365387	1050348	818 (0–3116)	98.99
616	14101949	9260787	1049327	938 (0–3397)	99.80
617	13494107	9514871	1051294	781(0-3674)	96.95
618	14998409	9704324	1049233	500 (0–2453)	91.91
619	7344494	5125076	1051007	500 (0–2230)	91.80
620	10438209	7264782	1049265	878 (0–3238)	99.80
621	13672749	9464302	1050293	529 (0–2887)	90.78
622	12884991	8588974	1050948	1150 (0–5200)	98.83
623	12545978	8817376	1050431	500 (0–3109)	90.11
624	10250460	6405329	1050282	1150 (0–5021)	97.62
625	17298286	12166173	1050302	1104 (0–5200)	99.80
626	7052465	4836179	1048892	500 (0–3109)	87.52
627	16158855	11497490	1051389	500 (0–2022)	92.92
628	11418087	7832824	1048790	704 (0–3514)	95.22
629	16938831	12452509	1049389	1150 (0–5200)	97.63
630	9987109	6509053	1050180	655 (0–3184)	93.26
631	11809067	8246062	1048549	663 (0–3078)	97.42
632	16777128	10962509	1049613	550 (0–2900)	90.15
633	12747489	8619959	1050329	791 (0–2924)	99.80
634	9114430	6400801	1049490	786 (0–3314)	98.99
635	15029829	10502287	1050166	1150 (0–4923)	98.88
636	15362471	11404886	1049515	1150 (0–3788)	99.03
637	16398000	10977769	1050424	775 (0–3072)	98.02
638	15245078	10954809	1049518	939 (0–5200)	94.76
639	10243956	6887739	1049536	941 (0–4707)	99.80
640	16486961	10864462	1049675	781 (0–2720)	99.66
641	9339988	5896441	1049613	941 (0–4707)	94.48
642	13495714	9242946	1049684	575 (0–2466)	94.09

643	7913921	5257699	1050550	668 (0–3320)	93.68
644	16811808	11859478	1050546	657 (0–2976)	93.20
645	16922347	11238943	1049636	582 (0–2187)	94.75
646	7351711	4894048	1050022	718 (0–4283)	90.66
647	7552289	5215910	1051235	500 (0–2018)	89.86
648	17169373	12561740	1050109	1069 (0–5200)	96.72
649	10997718	7119268	1050412	846 (0–3834)	95.49
650	13458415	9562586	1050347	610 (0–3414)	91.01
651	17006966	12010713	1050044	774 (0–4321)	96.40
652	13189267	8881278	1049791	999 (0–5001)	96.72
653	17428030	12204196	1049745	500 (0–2219)	91.89
654	14930361	10025315	1050486	688 (0–2829)	93.21
655	15134678	11351008	1049100	500 (0–2314)	88.57
656	12122599	8202601	1049833	500 (0–2166)	91.54
657	14211583	9698161	1050156	681 (0–3771)	92.70
658	9433095	6322308	1051233	850 (0–3484)	95.78
659	14441787	9086720	1051270	616 (0–2996)	92.13
660	12944321	8699948	1049523	606 (0–3107)	93.02
661	8779065	6030876	1050577	619 (0–2810)	91.71
662	14299075	10367225	1050004	736 (0–3989)	92.55
663	8350359	5801485	1050717	500 (0–1800)	87.74
664	12757170	8602396	1048863	500 (0–2413)	90.92
665	11118371	7580486	1050804	1150 (0–3450)	99.80
666	7062728	4872013	1049700	911 (0–3449)	97.09
667	15583327	10419496	1050265	619 (0–3581)	92.52
668	16376067	11470896	1050235	1150 (0–4579)	99.80
669	9179065	6689268	1048038	634 (0–2733)	95.36
670	7419608	4964501	1050199	715 (0–3554)	96.30
671	11501243	8237488	1049743	1009 (0–5200)	98.02
672	16568326	11702039	1050902	1041 (0–4367)	97.44
673	12685987	8830936	1050880	500 (0–2848)	89.85
674	12986875	8874682	1050153	567 (0–2969)	89.79
675	17882334	12341198	1049458	500 (0–2209)	90.09
676	12220756	8230413	1050606	892 (0–3497)	96.92
677	9447419	6944855	1051057	599 (0–2598)	92.76
678	7999375	5267172	1050488	858 (0–3598)	97.40

679	17289613	12345619	1050161	700 (0–3323)	95.33
680	11307928	8215115	1051135	500 (0–2395)	90.90
681	8249549	5529722	1050720	939 (0–3632)	99.65
682	11504933	8225744	1049182	579 (0–2983)	93.38
683	12132625	8302591	1049207	972 (0–3807)	98.90
684	14959959	10200759	1048394	1028 (0–4666)	94.87
685	17061511	11563662	1048213	1150 (0–3450)	99.80
686	15411372	9733639	1050593	1150 (0–3462)	98.75
687	14286753	9949834	1049476	683 (0–3793)	95.39
688	15167067	10970439	1050215	525 (0–2579)	91.20
689	9470999	6240047	1050638	611 (0–2822)	96.27
690	9283576	6548822	1049659	673 (0–3244)	94.79
691	7132777	4832056	1050386	625 (0–2026)	95.23
692	16250626	10517719	1049221	588 (0–3209)	91.06
693	15717451	11158395	1050564	1150 (0–4522)	99.80
694	10074372	7360146	1049828	1150 (0–5200)	98.77
695	8484882	5567546	1048941	630 (0–3392)	91.22
696	12807179	8791796	1050281	512 (0–2296)	91.97
697	11348762	7226982	1049161	690 (0–4021)	90.66
698	7606480	5162821	1051209	803 (0–4843)	94.04
699	16278555	11306791	1050687	799 (0–3929)	98.71
700	12001919	8792453	1050821	1131 (0–5200)	99.80
701	12393801	9006912	1050806	706 (0–3248)	95.47
702	9222440	6725694	1050545	1099 (0–5200)	97.04
703	13670667	9079883	1049943	748 (0–4536)	92.91
704	13096318	8809173	1049145	649 (0–3494)	93.86
705	13136298	8276798	1050060	855 (0–3254)	99.18
706	12413333	8174743	1050258	926 (0–4067)	97.68
707	17768305	11308551	1049517	1150 (0–5200)	96.38
708	12249103	8463715	1051267	526 (0–2407)	93.13
709	15416451	10160833	1049836	500 (0–2173)	89.85
710	9114592	5896752	1049769	749 (0–2982)	97.42
711	10683328	7150753	1049485	855 (0–2874)	99.80
712	13481486	8688193	1049491	564 (0–2641)	91.23
713	17127504	11805483	1050803	871 (0–4565)	95.67
714	13408633	9232292	1048323	521 (0–2716)	89.95

715	9522677	6385227	1050944	551 (0–2141)	93.23
716	17896753	11777490	1050504	1150 (0–4927)	95.38
717	11198738	7165140	1049719	583 (0–3097)	93.50
718	12016147	8371177	1049366	782 (0–2828)	97.39
719	7201942	4946738	1048992	858 (0–3383)	98.37
720	12913299	8960919	1050543	536 (0–2247)	92.12
721	10136185	6799423	1050082	861 (0–3501)	96.94
722	9975673	6718240	1050305	965 (0–5094)	96.01
723	11167780	8077208	1050485	538 (0–2612)	91.84
724	7724191	5113279	1049709	655 (0–2781)	93.09
725	17866359	11981550	1051159	959 (0–4645)	98.49
726	16602928	11660515	1050151	667 (0–2785)	93.30
727	14529867	10012347	1050584	604 (0–3133)	91.44
728	16591970	10263815	1049538	892(0-4217)	97.39
729	13830628	8704491	1050451	576 (0–3148)	89.84
730	13371869	8827774	1050446	1150 (0–5200)	99.80
731	10312345	7294014	1051241	824 (0–3195)	96.63
732	15613898	10932074	1050502	748 (0–4536)	92.30
733	12098427	8415493	1050742	1150 (0–4794)	99.42
734	7535945	5266362	1049530	500 (0–2220)	91.60
735	10048621	6540966	1050332	1061 (0–5200)	95.92
736	8326920	5812452	1048999	960 (0–3343)	99.80
737	8367094	5458370	1049896	918 (0–3548)	99.80
738	9031608	6398162	1050631	1150 (0–5200)	97.78
739	15712477	10443817	1048720	1062 (0–3981)	99.80
740	13743732	9461856	1050131	500 (0–2848)	87.61
741	11743956	7325469	1050463	621 (0–3033)	94.07
742	11477777	7340421	1050828	615 (0–2654)	92.58
743	13379283	9535687	1050708	500 (0–1800)	91.99
744	8453287	5923660	1049231	958 (0–4634)	97.46
745	12113295	7794444	1049971	1150 (0–5200)	99.13
746	10894640	7828537	1050954	1150 (0–4594)	99.80
747	9284322	6009523	1050907	807 (0–3497)	94.93
748	10809847	7357169	1050907	921 (0–3155)	99.80
749	12954525	8622493	1048873	1023 (0–4132)	98.68
750	7431030	4847243	1049289	500 (0–2324)	89.05

751	10046978	7183973	1048732	515 (0–1968)	91.21
752	16050038	11725346	1050056	964 (0–3865)	99.62
753	12287737	8421379	1048741	764 (0–4103)	96.77
754	13534361	9822968	1050231	1148 (0–5200)	98.70
755	17782020	11941756	1047902	506 (0–2445)	91.84
756	10894087	7443377	1050092	500 (0–2324)	88.08
757	12798508	8753471	1050205	500 (0–2395)	90.33
758	14925487	9631398	1051189	513 (0–2748)	90.83
759	9086627	6315058	1051003	646 (0–4022)	91.65
760	17080723	11978554	1051000	566 (0–3161)	90.43
761	10327249	6968784	1049739	607 (0–3430)	92.35
762	8306673	5226953	1050851	500 (0–2379)	89.06
763	17890195	12362682	1050415	500 (0–2067)	88.64
764	12978410	8235445	1049197	849 (0–4144)	93.38
765	14006944	9183820	1049757	607 (0–3430)	90.09
766	8904468	6090145	1050534	1150 (0–5200)	97.43
767	16964845	11144378	1050156	715 (0–3554)	94.26
768	10203095	7015753	1048705	832 (0–3176)	96.17
769	16766916	11287717	1050247	500 (0–1945)	92.52
770	8347485	5267973	1050084	733 (0–4077)	94.23
771	11019498	7548788	1050752	641 (0–2748)	91.94
772	11796006	8228863	1049168	802 (0–4293)	93.90
773	17900877	12716282	1051003	749 (0–2982)	97.54
774	7855459	5255325	1048685	737 (0–2512)	98.76
775	9868982	6692704	1049304	521 (0–1950)	92.60
776	13151948	8353417	1049140	711 (0–3619)	93.51
777	16195751	11967796	1050708	567 (0–2406)	92.72
778	9361689	6788631	1050908	706 (0–3248)	96.10
779	14321556	9724981	1048259	851 (0–4236)	99.00
780	14914282	10085854	1050712	500 (0–2567)	89.15
781	7657968	4594781	1050183	919 (0–4302)	96.50
782	10893422	7745854	1050178	500 (0–2253)	88.43
783	12535473	8735794	1050395	941 (0–3793)	97.89
784	7646290	5385927	1050002	798 (0–3536)	96.07
785	11595595	7744032	1049718	795 (0–2980)	94.99
786	8245475	5490803	1049755	527 (0–2321)	90.83

787	7573817	4869491	1049174	1022 (0–5200)	96.98
788	14202653	9989298	1050845	1026 (0–4594)	99.76
789	16971744	12178323	1049766	1084 (0–5200)	99.37
790	7778859	5551900	1050598	801 (0–3365)	97.82
791	17585470	12056910	1048718	1150 (0–5200)	99.80
792	9679613	6487281	1050921	969 (0–5200)	95.19
793	17176731	11138095	1049842	1004 (0–5200)	94.82
794	14402258	9490332	1050652	500 (0–2424)	88.78
795	8799268	6106925	1050451	1150 (0–4925)	99.14
796	14761438	9911765	1050084	685 (0–3565)	95.32
797	15743593	11480891	1049882	941 (0–4742)	94.88
798	14319398	10232222	1050764	555 (0–2969)	90.70
799	9400272	6222470	1050925	500 (0–2898)	88.66
800	8721051	5612259	1048554	722 (0–3617)	95.91
801	15435395	10242117	1048191	820 (0–3344)	94.89
802	14922450	10624210	1051221	687 (0–2622)	92.37
803	9787542	6501691	1050742	802 (0–3452)	96.75
804	7149031	4978440	1048549	500 (0–1909)	88.93
805	10210619	6769881	1050633	941 (0–5200)	98.71
806	10928105	7543615	1050382	952 (0–3498)	98.30
807	11695877	8138509	1049580	858 (0–4310)	98.08
808	15656023	11109122	1051134	1144 (0–5083)	99.45
809	11496021	8311591	1050896	858 (0–4310)	95.72
810	14305268	10289878	1050188	778 (0–2876)	99.59
811	10018452	6653202	1050642	1103 (0–5182)	96.56
812	9969933	6735581	1050069	1081 (0–5200)	96.09
813	7400280	4771658	1050161	871 (0–4565)	96.06
814	14889040	10019273	1050686	602 (0-3806)	90.68
815	10853426	7388247	1050890	733 (0–3381)	95.15
816	16003885	10688694	1050874	945 (0–5046)	96.70
817	15444471	10268912	1049807	985 (0–4173)	95.58
818	10216667	7263666	1050937	582 (0–2187)	92.72
819	13709953	9696014	1049105	724 (0–4344)	94.49
820	16235054	11234230	1050725	1150 (0–5200)	97.55
821	11376447	7299978	1048829	648 (0–2589)	96.08
822	7860602	5392592	1049218	947 (0–4214)	97.23

823	8040183	5374646	1050380	718 (0–3710)	96.45
824	12573653	9046389	1049733	618 (0–2901)	93.83
825	11223401	7025078	1050694	739 (0–2682)	95.16
826	8255811	5475545	1050085	742 (0–3049)	94.79
827	14429862	9558348	1049921	982 (0–4131)	96.13
828	17813045	11863537	1050180	949 (0–3968)	98.25
829	9993444	7082627	1051174	559 (0–1913)	96.14
830	9266664	5647376	1050426	796 (0–3533)	96.48
831	16356345	11020225	1050386	722 (0–3617)	94.72
832	14620670	10315199	1050039	1150 (0–4619)	98.65
833	14136582	10028541	1051147	750 (0–3460)	95.82
834	17793860	11806718	1050364	972 (0–3807)	99.80
835	8007706	5281936	1050506	536 (0–2247)	93.24
836	12338456	8518302	1050340	575 (0–2273)	92.79
837	12869072	8809280	1048032	794 (0–3840)	96.19
838	7284571	4968675	1049580	890 (0–3639)	99.80
839	17020762	10212457	1050670	1134 (0–5070)	99.80
840	9128308	5898574	1051108	500 (0–2898)	88.04
841	8974960	5870551	1049073	1069 (0–5200)	99.80
842	10714363	7492450	1050635	718 (0–4283)	91.03
843	16618000	10581964	1049870	977 (0–3925)	97.38
844	9056828	5874269	1049995	1018 (0–3492)	98.91
845	10382309	7011884	1050723	892(0-4217)	96.21
846	17579712	11275138	1049685	737 (0–2512)	98.83
847	7138987	4992191	1050904	646 (0–4022)	91.34
848	15397732	10289656	1050320	918 (0–3548)	98.17
849	10078887	7407853	1050057	781 (0–2720)	95.40
850	12055218	7969806	1050619	581 (0–1897)	93.58
851	7080748	4692493	1049314	890 (0–5200)	93.80
852	8565179	6031026	1049276	1150 (0–5200)	99.80
853	14196805	9374717	1050821	917 (0–4921)	94.47
854	8369213	6070584	1049519	840 (0–3615)	97.68
855	14392038	9995090	1049824	909 (0–4297)	95.98
856	7704393	5168751	1048488	1081 (0–5200)	97.88
857	12323888	8320794	1050276	918 (0–3548)	99.58
858	8580898	6435674	1050365	500 (0–2088)	90.17

859	14250454	9899215	1050471	1150 (0–5200)	95.85
860	14208048	9725612	1050605	1002 (0–3968)	97.64
861	17815302	12043549	1048584	816 (0–3251)	98.13
862	13087870	9306979	1050423	1150 (0–5200)	95.83
863	10589697	6718635	1049119	776 (0–3913)	92.22
864	9241171	6233765	1050423	514 (0–1860)	90.80
865	13491805	8095083	1050375	608 (0–2594)	91.33
866	9518744	6320425	1049209	922 (0–5200)	95.08
867	9519049	6715097	1050815	947 (0–4214)	97.00
868	12718715	9176132	1050660	1150 (0–5200)	97.70
869	15984656	11160153	1050428	1053 (0–5200)	97.39
870	12828488	8969667	1048218	675 (0–3428)	92.79
871	7903567	4967573	1049482	665 (0–3689)	93.99
872	10718411	7070387	1050987	949 (0–4455)	98.25
873	14128557	10232355	1050055	786 (0–3314)	95.13
874	9739856	6470032	1049729	871 (0–4565)	96.25
875	15757020	10650493	1050201	865 (0–4425)	94.75
876	8406434	5513218	1050988	1150 (0–5200)	98.67
877	14221443	9272202	1049927	500 (0–2512)	88.20
878	15526854	10339207	1049349	1044 (0–5200)	96.98
879	12269138	8099696	1051132	520 (0–2529)	87.24
880	9832878	6968807	1048301	1063 (0–4741)	99.22
881	8457691	5805876	1051278	748 (0–2904)	97.64
882	15615528	11095871	1050422	606 (0–3107)	92.06
883	8822933	5632409	1049797	688 (0–2829)	93.58
884	15462852	11083209	1050425	969 (0–5200)	99.19
885	16485420	11220697	1050683	519 (0–2560)	90.53
886	15145072	11134361	1050875	519 (0–2560)	92.54
887	17594783	11936595	1049363	878 (0–3238)	97.43
888	16406203	10421186	1050566	590 (0–2473)	92.28
889	12394899	8906065	1049238	912 (0–4262)	98.23
890	7035704	4755470	1050143	825 (0–4012)	94.09
891	15420651	9490432	1050543	778 (0–2876)	96.78
892	17478075	11961223	1049604	703 (0–3535)	94.91
893	10273867	7057498	1049966	702 (0–3171)	95.18
894	10000337	7123074	1050746	1150 (0–4799)	99.78

895	16670260	10002156	1051076	1033 (0–3992)	97.75
896	16195058	11570820	1050331	840 (0–3624)	95.55
897	11653761	7758632	1048849	1150 (0–4991)	95.87
898	11664108	8605632	1050367	778 (0–2334)	97.84
899	16862856	10597488	1050125	1023 (0–4132)	98.55
900	7377527	5137416	1050003	831 (0–3419)	97.19
901	16425835	11372453	1049989	538 (0–2612)	89.79
902	11397720	7606115	1050239	893 (0–4878)	95.96
903	9062649	5952219	1050469	648 (0–2693)	93.32
904	12879080	8613370	1050010	870 (0–3477)	99.80
905	8014419	5366442	1049958	950 (0–3549)	99.80
906	11528698	7405999	1050043	1150 (0–3450)	99.80
907	13178499	9353964	1049862	890 (0–4135)	97.93
908	10480436	6783294	1049166	565 (0–2284)	94.15
909	16899251	12010226	1050484	634 (0–2704)	95.14
910	17406227	10902076	1047692	1150 (0–4424)	99.80
911	12110054	8170463	1048605	1150 (0–4812)	98.90
912	16890193	11403090	1049448	575 (0–2273)	94.98
913	9145990	6214940	1049738	724 (0–2686)	97.97
914	10837890	7680692	1049287	628 (0–2951)	91.43
915	10206343	6878356	1049457	574 (0–2941)	90.69
916	10127751	6747737	1050370	1150 (0–4790)	98.80
917	7446592	5019153	1050579	659 (0–4046)	92.33
918	13809962	9797204	1051000	1150 (0–4646)	99.61
919	10164277	6544085	1050416	791 (0–3893)	90.91
920	12126356	7874612	1050180	1132 (0–5200)	97.86
921	8181926	5083423	1050017	715 (0–3734)	94.98
922	8727777	6465690	1050419	1104 (0–5200)	98.67
923	9576788	6179017	1050208	1150 (0–5200)	94.93
924	14796911	9224943	1050923	646 (0–3203)	95.60
925	8921184	5849915	1050182	550 (0–2209)	95.32
926	12048183	7971395	1051203	594(0-4633)	89.48
927	10313766	7466792	1049812	1133 (0–5200)	97.57
928	12220280	8139354	1051250	763 (0–3944)	92.94
929	7738830	5330143	1048468	548 (0–1813)	95.11
930	7123336	4461179	1050453	710 (0–3663)	96.24

931	10494557	7201780	1049715	677 (0–3265)	94.61
932	9431602	5946966	1050762	909 (0–3978)	95.10
933	15411387	10334755	1049297	500 (0–2354)	88.84
934	13649647	8970601	1048652	906 (0–3121)	99.80
935	10794772	7359316	1050174	890 (0–3639)	99.17
936	12771260	8927523	1049043	860 (0–4994)	96.12
937	15523977	9934152	1049293	908 (0–4374)	97.00
938	7458953	4817793	1049744	1024 (0–4029)	97.29
939	17618392	12082828	1049665	1150 (0–5200)	97.80
940	17783247	12459273	1051218	840 (0–3624)	98.78
941	11844645	8558008	1050626	875 (0–4910)	95.35
942	9444478	6288542	1050684	608 (0–2129)	94.25
943	12292083	7808615	1049850	1015 (0–4082)	98.45
944	17706200	12139349	1050255	1150 (0–5200)	98.80
945	17998156	11809682	1050188	807 (0–4216)	96.76
946	10698026	6977974	1050602	944 (0–3704)	97.92
947	14632463	10568228	1049184	520 (0–2618)	89.56
948	7646513	5341560	1050432	500 (0–2253)	88.54
949	8903464	5859883	1049864	890 (0–5200)	93.41
950	13761479	9350081	1050637	1043 (0–5200)	97.82
951	12687710	8595031	1051071	645 (0–2585)	96.41
952	17553333	12023206	1050594	520 (0–2529)	91.42
953	16250409	10852378	1049786	806 (0–3653)	97.97
954	13670653	8516791	1048632	906 (0–4465)	97.36
955	15261537	9930827	1051163	1139 (0–4407)	99.80
956	7079611	4701146	1049385	677 (0–3265)	93.57
957	14537619	9897841	1050045	702 (0–3171)	95.72
958	8414084	5448382	1048831	813 (0–3774)	95.72
959	12549146	8603551	1049216	1150 (0–4669)	99.40
960	17429028	11652383	1048656	1132 (0–4450)	99.80
961	9628900	6328265	1051038	782 (0–3595)	95.69
962	12368862	7939470	1050755	810 (0–3571)	97.51
963	7976032	5530539	1050554	839 (0–3946)	97.97
964	17561119	12165067	1050999	781 (0–2532)	99.28
965	15831824	11145709	1048642	1133 (0–5200)	99.80
966	13056943	9093882	1050909	708 (0–3587)	94.01

967	7012328	4899682	1049976	880 (0–3191)	98.22
968	8078500	5585979	1050458	690 (0–4021)	92.36
969	9084602	5698296	1050289	1010 (0–5200)	96.98
970	15868728	10657857	1050536	676 (0–3394)	93.80
971	7978512	5082540	1050433	975 (0–3750)	99.80
972	15988271	11528764	1049625	500 (0–1952)	90.38
973	15277521	10382291	1050438	709 (0–2821)	97.52
974	7692109	5523647	1050617	691 (0–3195)	93.79
975	13428559	8716348	1049955	551 (0–2141)	90.86
976	12103078	8196736	1050431	891 (0–4808)	96.58
977	12916354	8885176	1049285	750 (0–3460)	95.11
978	16334566	10945351	1049547	839 (0–3946)	96.58
979	17674748	12118403	1050862	591 (0–2534)	95.36
980	8126095	5887071	1048747	1054 (0–5200)	96.14
981	13735131	8914685	1050523	917 (0–5200)	92.25
982	14922055	8966101	1051125	594(0-4633)	88.33
983	8101198	5897824	1051034	676 (0–3364)	95.74
984	16264629	10673665	1049132	1150 (0–4403)	99.80
985	16169096	11539479	1050189	923 (0–3304)	99.80
986	14604113	10216415	1049745	1150 (0–5200)	98.77
987	11157100	7514300	1048355	905 (0–3752)	99.80
988	13896698	9608321	1050882	500 (0–2379)	92.06
989	10228395	6605334	1051218	1150 (0–4533)	98.95
990	10467201	7144670	1050995	837 (0–4096)	96.18
991	10965861	7652210	1050200	1150 (0–4594)	99.80
992	15185733	9973534	1051233	500 (0–2608)	88.78
993	8101270	5416438	1050512	500 (0–2113)	92.45
994	7261721	5006069	1050737	634 (0–2733)	96.21
995	11213241	8070149	1050185	1150 (0–4279)	99.51
996	7604949	5170646	1050807	500 (0–2068)	90.50
997	14755859	9843341	1050893	767 (0–2914)	96.75
998	14314717	9671131	1049739	776 (0–4022)	95.03
999	17258427	12336415	1048949	932 (0–3470)	97.96
1000	7224671	4773735	1049874	956 (0–5200)	94.22
1001	11097830	7446567	1050086	874 (0–4333)	94.47
1002	8357669	5524688	1050076	500 (0–2417)	88.64

1003	15420637	10391512	1050315	974 (0–3663)	97.50
1004	11739866	8058372	1050799	608 (0–2129)	96.28
1005	17467851	12419928	1049651	917 (0–5200)	94.15
1006	14727021	9075714	1049966	1139 (0–4407)	99.56
1007	14608790	10177428	1049543	821(0-4675)	95.65
1008	17553166	11466646	1050149	500 (0–1800)	92.85
1009	17143330	11962480	1050374	643 (0–2544)	98.35
1010	15992232	11469454	1050977	789 (0–3608)	97.45
1011	16746737	11887970	1049451	1045 (0–5172)	98.39
1012	16932010	11594004	1051085	1150 (0–5200)	97.88
1013	12669826	8932670	1050229	500 (0–2415)	90.61
1014	14432014	9405322	1050858	795 (0–2980)	97.83
1015	17433092	11819028	1050790	583 (0–3322)	91.45
1016	8780315	6370857	1049334	537 (0–2734)	90.34
1017	15394151	10103525	1049197	1044 (0–5200)	97.81
1018	7630409	5233835	1051042	719 (0–3035)	94.59
1019	7640685	5111043	1050312	802 (0–4293)	95.63
1020	15540117	10476319	1050279	1001 (0–5095)	96.56
1021	16555330	11459304	1050301	774 (0–4321)	93.08
1022	16971889	11403657	1050330	738 (0–3708)	95.82
1023	12291814	8357176	1050666	686 (0–3404)	95.70
1024	14467749	9619064	1049306	642 (0–3582)	95.45
1025	14458915	9040424	1049356	958 (0–5200)	97.65
1026	15108971	10361624	1050948	883 (0–4701)	94.55
1027	9967361	6909216	1050793	766 (0–3295)	97.30
1028	17219701	12423415	1049056	610 (0–3414)	91.19
1029	17987448	12160307	1050922	735 (0–3535)	95.08
1030	15627928	9973099	1049036	526 (0–1822)	88.90
1031	14707813	9668618	1051118	500 (0–1952)	92.30
1032	15283461	10640525	1047874	500 (0–2278)	90.63
1033	9022094	6112145	1049803	871 (0–4565)	99.16
1034	9547348	6572191	1050584	625 (0–3241)	91.87
1035	15224825	10191614	1051082	1150 (0–4927)	97.84
1036	7720979	5683368	1049648	924 (0–3527)	98.86
1037	9046348	6620476	1049614	500 (0–1800)	90.88
1038	13354506	8707834	1050907	936 (0–3695)	98.70

1039	12034051	8511334	1050266	500 (0–1909)	89.77
1040	14312052	9862748	1049989	844 (0–3840)	96.91
1041	17415521	11849639	1050366	640 (0–3327)	95.31
1042	12033785	7872615	1049520	1150 (0–5200)	99.66
1043	7192677	5240915	1050701	918 (0–4115)	98.22
1044	17291255	12362517	1050497	858 (0–3598)	99.60
1045	15408461	9804203	1050722	1011 (0–5200)	99.80
1046	9443608	6813608	1048978	502 (0–1941)	88.03
1047	8638674	5864352	1048046	526 (0–2407)	90.29
1048	10356795	6856885	1050044	611 (0–2822)	94.30
1049	16660024	10840722	1050997	1024 (0–4074)	97.93
1050	13263889	9395404	1050837	775 (0–2325)	98.06
1051	17533622	12133211	1048569	1150 (0–5200)	96.97
1052	10072240	6584985	1047466	741 (0–2807)	97.87
1053	16057611	10528447	1051256	906 (0–4844)	96.69
1054	14454436	9660974	1050182	1150 (0–5103)	99.80
1055	13635445	9062455	1050376	903 (0–4007)	99.80
1056	12114696	8429810	1050453	500 (0–1987)	92.00
1057	10325979	7138991	1048832	500 (0–2159)	94.90
1058	15507093	10249111	1050617	1004 (0–5200)	94.70
1059	9968448	6282861	1050025	720 (0–4240)	91.51
1060	11318091	7243352	1048006	642 (0–3582)	92.00
1061	12157955	8584752	1048907	500 (0–2270)	89.25
1062	8727959	5674532	1048674	774 (0–3726)	95.96
1063	12766590	8723846	1049347	778 (0–2334)	99.44
1064	7335292	4600000	1050016	599 (0–2939)	93.25
1065	12656570	8757230	1050039	809 (0–3812)	99.36
1066	17427269	12854106	1048459	1099 (0–5200)	95.27
1067	11279936	7220941	1050804	1133 (0–5200)	99.00
1068	11593360	8074670	1050838	500 (0–2159)	91.72
1069	11288130	7768196	1048267	829 (0–3866)	95.97
1070	15314034	10835238	1050850	828 (0–3840)	94.07
1071	9002946	6086557	1050197	634 (0–2733)	95.63
1072	7928631	5456363	1050202	863 (0–5155)	96.35
1073	17123912	11737743	1049601	709 (0–3833)	95.01
1074	8173649	5698548	1051369	599 (0–2776)	93.49

1075	10476117	6860040	1050403	676 (0–3364)	93.17
1076	16653755	11420703	1050807	1068 (0–4599)	96.10
1077	17196728	12111824	1049724	697 (0–2979)	96.51
1078	7821074	5592379	1049739	500 (0–2861)	86.33
1079	12976389	9016928	1050628	1150 (0–5200)	96.78
1080	7260925	4690857	1050306	1150 (0–4646)	99.80
1081	12311468	7797993	1048905	571 (0–2698)	92.08
1082	11129294	7704351	1049502	778 (0–2334)	99.64
1083	17687379	11490618	1050740	1150 (0–5200)	98.86
1084	9879477	6968292	1049507	772 (0–4846)	94.11
1085	14043768	9428515	1050353	645 (0–3114)	94.78
1086	11440740	8126912	1049685	500 (0–1987)	89.44
1087	17270425	12845302	1050165	711 (0–3367)	93.83
1088	10119123	6862084	1050411	975 (0–4826)	95.74
1089	13936198	9428117	1049282	611 (0–2555)	93.90
1090	15643762	11113403	1049350	1069 (0–5200)	95.48
1091	12736505	9067336	1050891	1150 (0–4800)	99.80
1092	8302536	6069554	1050404	1025 (0–4461)	98.15
1093	13883360	9522556	1050160	1150 (0–5200)	95.94
1094	17049237	11269784	1050157	1007 (0–4525)	99.80
1095	16052608	10919488	1049298	932 (0–3470)	96.55
1096	13425735	8878319	1050654	702 (0–3171)	96.96
1097	14162742	9732555	1050853	693 (0–2842)	95.40
1098	8994465	5881956	1050047	500 (0–2852)	88.22
1099	12024559	8502087	1049717	1034 (0–5200)	96.78
1100	13391870	9386693	1050572	756 (0–3033)	97.71
1101	17284919	11825840	1050169	781 (0–2532)	99.26
1102	16602288	11302295	1047810	524 (0–1800)	92.17
1103	13226296	8915935	1048498	938 (0–4510)	97.09
1104	8720138	6272985	1049889	807 (0–4231)	98.91
1105	7974930	5707040	1050367	564 (0–3264)	91.00
1106	9494728	6497921	1049770	659 (0–2457)	94.85
1107	14910616	9756024	1049594	1150 (0–5200)	99.80
1108	7485012	4825546	1050445	707 (0–2859)	94.82
1109	12684938	8735211	1048298	615 (0–2654)	96.56
1110	15415115	10187204	1050261	850 (0–3359)	99.79

1111	12147569	8433790	1049302	827 (0–3645)	96.14
1112	11567502	7731708	1051179	1150 (0–4770)	98.78
1113	14775749	10390493	1051031	616 (0–2339)	96.87
1114	7998800	5488956	1050858	785 (0–3206)	97.86
1115	13680376	9712268	1050399	865 (0–2595)	99.80
1116	15851780	9716869	1050134	791 (0–4119)	97.48
1117	12791967	8611405	1051126	961 (0–4573)	97.19
1118	15175471	11328544	1049327	947 (0–4214)	97.92
1119	13217394	7995270	1050503	953 (0–4294)	98.59
1120	13048322	9202715	1050059	628 (0–2951)	91.55
1121	15894158	11007342	1050364	723 (0–3470)	94.48
1122	10670191	7667290	1049215	1067 (0–5200)	96.77
1123	10536991	7110280	1049359	657 (0–2281)	97.76
1124	15358727	10992404	1049600	984 (0–4405)	99.80
1125	17346117	12428617	1050007	1150 (0–5200)	99.45
1126	10232772	7018693	1050495	724 (0–2686)	95.95
1127	7788334	5024096	1050372	1150 (0–4066)	99.80
1128	17925293	12680175	1050808	543 (0–2182)	90.76
1129	10695668	6960707	1050383	1150 (0–4281)	99.80
1130	10827472	7318058	1050345	898 (0–4574)	95.83
1131	9885508	7002966	1051049	845 (0–4313)	96.48
1132	10080026	7184660	1050205	800 (0–3407)	96.40
1133	14960084	10016692	1047340	516 (0–1965)	91.15
1134	11726445	8194551	1050005	515 (0–1968)	91.31
1135	7965665	5048032	1051007	500 (0–2279)	90.23
1136	15254414	10156825	1049877	798 (0–3717)	95.60
1137	16818402	12177659	1049893	710 (0–3596)	95.61
1138	14446809	10406060	1049867	1035 (0–3886)	99.76
1139	11956962	8370787	1050333	616 (0–3070)	92.61
1140	11815682	7984388	1051146	1150 (0–5200)	97.61
1141	7264616	5124590	1049566	655 (0–3184)	93.86
1142	10488136	7124624	1049867	802 (0–4293)	93.39
1143	15958115	10932057	1049996	810 (0–3571)	96.03
1144	7068019	4824994	1051033	961 (0–4305)	95.66
1145	17356153	11398383	1050575	500 (0–2478)	91.70
1146	9323417	6542967	1050916	538 (0–2332)	90.24

1147	7408073	4799702	1050291	558 (0–2034)	92.63
1148	9848745	6926380	1051174	829 (0–3327)	99.80
1149	10581881	6890730	1050379	1014 (0–3380)	99.80
1150	14951576	9843187	1049698	521 (0–2716)	88.89
1151	15207095	9124257	1049645	500 (0–2453)	89.81
1152	16873006	11394636	1050651	500 (0–2417)	91.49
1153	13582686	9313317	1050636	781(0-3674)	97.19
1154	17652235	12441510	1050428	607 (0–3741)	89.82
1155	17348696	12479373	1050894	500 (0–2067)	91.75
1156	17666039	11223121	1050085	599 (0–2939)	92.22
1157	7171165	5088263	1050203	625 (0–2082)	95.68
1158	11649218	7759299	1050848	723 (0–2942)	96.11
1159	7917493	5366779	1049879	1014 (0–3380)	99.80
1160	11973086	8103887	1050829	728 (0–3639)	94.21
1161	7976084	5316176	1050612	551 (0–2141)	93.60
1162	12792418	8357321	1051280	589 (0–2905)	90.41
1163	15797047	10874316	1049954	1150 (0–4319)	97.16
1164	9930268	6994229	1050786	634 (0–2733)	96.86
1165	10694732	6868230	1051197	500 (0–1800)	92.73
1166	16144093	10968559	1049060	583 (0–3322)	91.83
1167	16216453	10721178	1048723	583 (0–3097)	92.78
1168	8336458	5942875	1050480	516 (0–1965)	92.70
1169	15136458	10516923	1050920	887 (0–3907)	96.50
1170	12193283	8843314	1049409	1084 (0–5200)	98.61
1171	9638065	6106071	1048795	611 (0–3051)	91.72
1172	8471893	6023735	1051311	1139 (0–5200)	99.32
1173	17734945	12146308	1049938	879 (0–4999)	93.69
1174	16475549	10844944	1050905	896 (0–4550)	97.07
1175	12628318	8772591	1050723	500 (0–1800)	89.43
1176	17991106	13079192	1049584	500 (0–2763)	90.41
1177	17313275	12606461	1049390	555 (0–2160)	93.41
1178	7139785	5031168	1049517	500 (0–1800)	90.96
1179	14269018	9278455	1050773	642 (0–3655)	93.46
1180	8114980	5593622	1048840	567 (0–2406)	94.16
1181	15817494	10918384	1050828	589 (0–2266)	95.07
1182	13041173	9353918	1050136	984 (0–4405)	96.80

1183	14097814	9516782	1048611	941 (0–3793)	98.47
1184	12588878	8566318	1050449	958 (0–5200)	95.82
1185	9820194	6485204	1050961	543 (0–2182)	93.71
1186	8488356	5942211	1050713	710 (0–2852)	95.30
1187	8936131	6018821	1049519	775 (0–2325)	98.38
1188	10260621	7034470	1049622	961 (0–4305)	99.80
1189	9588257	6384950	1048871	850 (0–3484)	96.44
1190	16086471	10961598	1050831	654 (0–1962)	96.54
1191	9001912	5829527	1051095	906 (0–4844)	97.36
1192	13846461	9482426	1050230	866 (0–4101)	95.32
1193	11716890	7755676	1050300	555 (0–2969)	94.20
1194	8493578	5953827	1049238	840 (0–3624)	95.36
1195	12513018	9202369	1050660	1150 (0–5200)	97.98
1196	11308026	7976354	1051136	500 (0–2146)	91.03
1197	15083984	9750322	1050647	675 (0–3428)	95.46
1198	9208121	6162269	1049559	910 (0–4158)	96.74
1199	7571805	4783178	1050927	689 (0–3505)	92.25
1200	7449330	5068577	1049795	561 (0–2346)	92.75
1201	10653093	6677074	1050284	598 (0–3086)	94.68
1202	12320248	7933731	1049926	875 (0–4293)	94.26
1203	16469943	11741220	1051217	863 (0–3252)	98.62
1204	17435959	12306842	1049424	500 (0–2633)	91.64
1205	9858785	6254305	1050770	500 (0–2616)	87.88
1206	8481974	5653887	1050858	811 (0–3221)	97.92
1207	9756437	6770037	1050068	1084 (0–5200)	96.42
1208	10827199	7489983	1049710	500 (0–2415)	90.40
1209	14995800	9465124	1050884	823 (0–3827)	96.49
1210	7517261	5170374	1050522	561 (0–3091)	86.80
1211	16608856	10991150	1050854	645 (0–2434)	97.03
1212	11188522	8010535	1050303	536 (0–2637)	91.90
1213	9819393	6618218	1050326	754 (0–4340)	94.90
1214	14687252	10145640	1050026	500 (0–2075)	93.29
1215	10476783	7089915	1050356	761 (0–3352)	97.94
1216	11537651	8076279	1050691	1132 (0–4023)	99.80
1217	14050079	9230311	1050670	918 (0–3548)	99.03
1218	7053603	4937858	1050142	638 (0–3363)	92.60

1219	7427444	5320047	1050760	1150 (0–4876)	97.42
1220	9648538	7092320	1049223	1150 (0–4388)	99.80
1221	7459034	5426549	1050195	1150 (0–5140)	97.54
1222	10068521	6684656	1049245	554 (0–2742)	92.35
1223	12655446	8824826	1049391	632 (0–2746)	93.35
1224	12559484	8844702	1050205	653 (0–3230)	93.12
1225	9699415	6767295	1050116	636 (0–3273)	93.56
1226	14447652	10150983	1049237	923 (0–3304)	99.80
1227	11554515	8004024	1051267	936 (0–3790)	99.73
1228	15081068	10008435	1051154	737 (0–3238)	94.00
1229	8852132	6074451	1050919	641 (0–2838)	90.40
1230	15559478	10975712	1050875	1097 (0–4999)	97.40
1231	17348655	12037332	1050225	669 (0–3374)	95.27
1232	14804112	9579880	1050847	500 (0–3068)	86.71
1233	13571129	8871060	1051120	1064 (0–5200)	97.84
1234	9644384	6439775	1050475	667 (0–2785)	95.65
1235	9523760	6417639	1048961	952 (0–4342)	99.75
1236	13950004	9551822	1050153	623 (0–3410)	93.83
1237	13748128	8945730	1050871	710 (0–2852)	93.94
1238	13393260	9426828	1050646	540 (0–2882)	89.71
1239	9740109	6080488	1049479	799 (0–3929)	96.91
1240	11375769	7915955	1050774	642 (0–2845)	92.11
1241	17481657	12153178	1049991	1150 (0–4927)	98.01
1242	13656727	9550463	1049232	761 (0–3352)	99.45
1243	17102283	10261370	1049752	638 (0–3326)	93.69
1244	13362802	8545802	1050564	932 (0–4696)	94.58
1245	17959432	12022387	1049980	975 (0–4826)	96.07
1246	9775684	6696817	1050197	500 (0–2567)	90.14
1247	11809291	8001483	1049335	1119 (0–5049)	98.37
1248	12914796	8466780	1048726	895 (0–3515)	98.24
1249	13860426	9958596	1051052	1097 (0–4999)	99.51
1250	16252699	11209826	1050458	999 (0–4170)	98.99
1251	11161149	7457404	1050296	831 (0–4101)	95.61
1252	7949786	5170325	1049949	777 (0–3502)	96.16
1253	16910997	11534085	1049429	645 (0–2434)	95.72
1254	11356866	8151516	1050302	748 (0–2904)	96.15

1255	16343003	11130490	1049902	974 (0–3663)	99.80
1256	15713116	10669812	1050099	1150 (0–5200)	99.80
1257	7973257	5437669	1049706	932 (0–3470)	99.10
1258	9682830	6619361	1050842	908 (0–4374)	97.90
1259	17397296	11708457	1049491	583 (0–3322)	88.65
1260	10744895	6992932	1050261	1150 (0–3998)	99.80
1261	12683612	9206659	1050187	500 (0–2352)	90.37
1262	8586755	6003213	1050607	1150 (0–4619)	98.35

Table S3. Detailed *BRCA1/2* P/LP variants identified in the study cohort.

labID	variants	variants_type
18-CR-061	BRCA2 c.3596_3599del	frameshift_deletion
18-CR-062	BRCA2 c.3189_3192del	frameshift_deletion
18-CR-063	BRCA1 c.2110_2111del	frameshift_deletion
18-CR-087	BRCA1 c.4065_4068del	frameshift_deletion
18-CR-088	BRCA1 c.2110_2111del	frameshift_deletion
18-CR-097	BRCA1 c.5533_5540del	frameshift_deletion
18-CR-101	BRCA1 c.5530+1G>A	splicing
18-CR-127	BRCA1 c.5362del	frameshift_deletion
18-CR-128	BRCA2 c.7673_7674del	frameshift_deletion
18-CR-134	BRCA1 c.5533_5540del	frameshift_deletion
18-CR-146	BRCA2 c.793+1G>T	splicing
18-CR-164	BRCA1 c.302-2A>C	splicing
18-CR-171	BRCA2 c.9097del	frameshift_deletion
18-CR-277	BRCA2 c.476-2A>G	splicing
18-CR-313	BRCA1 c.981_982del	frameshift_deletion
18-CR-318	BRCA1 c.5533_5540del	frameshift_deletion
18-CR-357	BRCA2 c.3109C>T	stopgain
18-CR-368	BRCA1 c.3770_3771del	frameshift_deletion
18-CR-385	BRCA1 c.5533_5540del	frameshift_deletion
18-CR-392	BRCA1 c.3770_3771del	frameshift_deletion
18-CR-411	BRCA2 c.3631G>T	stopgain
18-CR-420	BRCA1 c.3770_3771del	frameshift_deletion
18-CR-422	BRCA1 c.4547+1G>T	splicing
18-CR-424	BRCA2 c.5164_5165del	frameshift_deletion
18-CR-441	BRCA1 c.5470_5477del	frameshift_deletion
18-CR-448	BRCA2 c.5164_5165del	frameshift_deletion
18-CR-468	BRCA2 c.3109C>T	stopgain
18-CR-491	BRCA2 c.1773_1776del	frameshift_deletion
18-CR-506	BRCA1 c.3770_3771del	frameshift_deletion
18-CR-510	BRCA1 c.303T>G	stopgain
18-CR-522	BRCA1 c.66dupA	frameshift_insertion
18-CR-528	BRCA2 c.4171del	frameshift_deletion
18-CR-538	BRCA1 c.1504_1508del	frameshift_deletion
18-CR-559	BRCA1 c.3756_3759del	frameshift_deletion
18-CR-566	BRCA1 c.2269del	frameshift_deletion
18-CR-572	BRCA2 c.476-3C>A	intronic
18-CR-575	BRCA2 c.2025_2026del	frameshift_deletion
18-CR-579	BRCA2 c.8385del	frameshift_deletion
18-CR-596	BRCA2 c.476-2A>G	splicing
18-CR-598	BRCA1 c.2800C>T	stopgain
18-CR-602	BRCA2 c.5645C>A	stopgain
18-CR-605	BRCA2 c.5164_5165del	frameshift_deletion
18-CR-611	BRCA2 c.3109C>T	stopgain
18-CR-614	BRCA1 c.5470_5477del	frameshift_deletion
18-CR-618	BRCA1 c.5470_5477del	frameshift_deletion
18-CR-621	BRCA1 c.5075-1G>A	splicing
18-CR-640	BRCA1 c.4573C>T	stopgain
18-CR-653	BRCA1 c.2296_2297del	frameshift_deletion
18-CR-658	BRCA2 c.7976+2C>G	splicing
18-CR-671	BRCA1 c.981_982del	frameshift_deletion
18-CR-676	BRCA2 c.5682C>G	stopgain

18-CR-692	BRCA2	c.9382C>T	stopgain
18-CR-707	BRCA2	c.6553del	frameshift_deletion
19-CR-001	BRCA1	c.5470_5477del	frameshift_deletion
19-CR-014	BRCA1	c.2110_2111del	frameshift_deletion
19-CR-015	BRCA2	c.196C>T	stopgain
19-CR-060	BRCA2	c.7409dupT	frameshift_insertion
19-CR-063	BRCA1	c.4573C>T	stopgain
19-CR-064	BRCA1	c.5277+1G>A	splicing
19-CR-068	BRCA1	c.1115G>A	stopgain
19-CR-075	BRCA1	c.5470_5477del	frameshift_deletion
19-CR-083	BRCA2	c.9117G>A	synonymous_SNV
19-CR-084	BRCA2	c.632-1G>C	splicing
19-CR-091	BRCA2	c.67+2T>C	splicing
19-CR-100	BRCA1	c.1116G>A	stopgain
19-CR-103	BRCA1	c.3607C>T	stopgain
19-CR-104	BRCA2	c.2808_2811del	frameshift_deletion
19-CR-107	BRCA1	c.1945G>T	stopgain
19-CR-112	BRCA1	c.5503C>T	stopgain
19-CR-119	BRCA1	c.4484+1G>T	splicing
19-CR-120	BRCA1	c.4065_4068del	frameshift_deletion
19-CR-121	BRCA1	c.2110_2111del	frameshift_deletion
19-CR-122	BRCA1	c.5470_5477del	frameshift_deletion
19-CR-124	BRCA1	c.5470_5477del	frameshift_deletion
19-CR-141	BRCA1	c.1058G>A	stopgain
19-CR-163	BRCA1	c.4484G>C	nonsynonymous_SNV
19-CR-172	BRCA2	c.1813del	frameshift_deletion
19-CR-180	BRCA2	c.3847_3848del	frameshift_deletion
19-CR-195	BRCA1	c.5521del	frameshift_deletion
19-CR-197	BRCA2	c.5722_5723del	frameshift_deletion
19-CR-203	BRCA1	c.5470_5477del	frameshift_deletion
19-CR-207	BRCA2	c.7976+2C>G	splicing
19-CR-214	BRCA1	c.5470_5477del	frameshift_deletion
19-CR-225	BRCA1	c.5407-1G>A	splicing
19-CR-248	BRCA1	c.5521del	frameshift_deletion
19-CR-249	BRCA2	c.4877dupA	frameshift_insertion
19-CR-271	BRCA2	c.818C>A	stopgain
19-CR-316	BRCA2	c.6478C>T	stopgain
19-CR-328	BRCA1	c.5503C>T	stopgain
19-CR-335	BRCA1	c.4258C>T	stopgain
19-CR-335	BRCA1	c.668del	frameshift_deletion
19-CR-353	BRCA2	c.5595_5596del	frameshift_deletion
19-CR-357	BRCA2	c.3847_3848del	frameshift_deletion
19-CR-371	BRCA1	c.5353C>T	stopgain
19-CR-375	BRCA2	c.3109C>T	stopgain
19-CR-377	BRCA1	c.5470_5477del	frameshift_deletion
19-CR-378	BRCA1	c.981_982del	frameshift_deletion
20-CR-001	BRCA1	c.4755del	frameshift_deletion
20-CR-016	BRCA2	c.5682C>G	stopgain
20-CR-018	BRCA2	c.6410del	frameshift_deletion
20-CR-027	BRCA1	c.5470_5477del	frameshift_deletion
20-CR-029	BRCA2	c.5682C>G	stopgain
20-CR-078	BRCA2	c.6405_6409del	frameshift_deletion
20-CR-082	BRCA1	c.5074G>A	nonsynonymous_SNV
20-CR-094	BRCA1	c.5467+1G>A	splicing

20-CR-096	BRCA2	c.7673_7674del	frameshift_deletion
20-CR-113	BRCA2	c.1310_1313del	frameshift_deletion
20-CR-126	BRCA2	c.8400_8402del	frameshift_substitution
20-CR-141	BRCA2	c.172G>T	stopgain
20-CR-169	BRCA1	c.1504_1508del	frameshift_deletion
20-CR-187	BRCA1	c.2361del	frameshift_deletion
20-CR-188	BRCA1	c.4986+1G>T	splicing
20-CR-207	BRCA1	c.3767_3768del	frameshift_deletion
20-CR-217	BRCA1	c.5074G>A	nonsynonymous_SNV
20-CR-220	BRCA1	c.981_982del	frameshift_deletion
20-CR-221	BRCA2	c.9382C>T	stopgain
20-CR-224	BRCA2	c.6486_6489del	frameshift_deletion
20-CR-228	BRCA1	c.2338C>T	stopgain
20-CR-233	BRCA2	c.6405_6409del	frameshift_deletion
20-CR-250	BRCA1	c.4372C>T	stopgain
20-CR-261	BRCA2	c.2808_2811del	frameshift_deletion
20-CR-262	BRCA2	c.2808_2811del	frameshift_deletion
20-CR-271	BRCA1	c.5470_5477del	frameshift_deletion
20-CR-289	BRCA1	c.2157dupA	frameshift_insertion
20-CR-290	BRCA2	c.610del	frameshift_deletion
